# Seasonal variation of two floral patterns in *Clematis* ‘Vyvyan Pennell’ and its underlying mechanism

**DOI:** 10.1186/s12870-023-04696-9

**Published:** 2024-01-02

**Authors:** Ying Wang, Yue Pan, Lei Peng, Jin Wang

**Affiliations:** 1 College of Landscape Architecture and Horticulture Science, Southwest Research Center for Engineering Technology of Landscape Architecture (State Forestry and Grassland Administration), Yunnan Engineering Research Center for Functional Flower Resources and Industrialization, Research and Development Center of Landscape Plants and Horticulture Flowers, Southwest Forestry University, Kunming, 650224 China Yunnan; 2 College of Horticulture and Landscape, Yunnan Agricultural University, Kunming, Yunnan 650201 China

**Keywords:** *Clematis* 'Vyvyan Pennell', Double-perianth, Stamen petaloidy, Floral pattern variation, Flower diversity

## Abstract

**Background:**

Floral patterns are crucial for insect pollination and plant reproduction. Generally, once these patterns are established, they exhibit minimal changes under natural circumstances. However, the *Clematis* cultivar’ Vyvyan Pennell’, the apetalous lineage in the Ranunculaceae family, produces two distinct types of flowers during different seasons. The regulatory mechanism responsible for this phenomenon remains largely unknown. In this study, we aim to shed light on this floral development with shifting seasonal patterns by conducting extensive morphological, transcriptomic, and hormone metabolic analyses. Our findings are anticipated to contribute valuable insights into the diversity of flowers in the Ranunculaceae family.

**Results:**

The morphological analysis revealed that the presence of extra petaloid structures in the spring double perianth was a result of the transformation of stamens covered with trichomes during the 5th developmental stage. A de novo reference transcriptome was constructed by comparing buds and organs within double and single perianth from both seasons. A total of 209,056 unigenes were assembled, and 5826 genes were successfully annotated in all six databases. Among the 69,888 differentially expressed genes from the comparative analysis, 48 genes of utmost significance were identified. These critical genes are associated with various aspects of floral development. Interestingly, the A-, B-, and C-class genes exhibited a wider range of expression and were distinct within two seasons. The determination of floral organ identity was attributed to the collaborative functioning of all the three classes genes, aligning with a modified “fading border model”. The phytohormones GA3, salicylic acid, and trans-zeatin riboside may affect the formation of the spring double perianth, whereas GA7 and abscisic acid may affect single flowers in autumn.

**Conclusions:**

We presumed that the varying temperatures between the two seasons served as the primary factor in the alteration of floral patterns, potentially affecting the levels of plant hormones and expressions of organ identity genes. However, a more thorough investigation is necessary to fully comprehend the entire regulatory network. Nonetheless, our study provides some valuable informations for understanding the underlying mechanism of floral pattern alterations in *Clematis*.

**Supplementary Information:**

The online version contains supplementary material available at 10.1186/s12870-023-04696-9.

## Introduction

Seasonal variations in angiosperm floral patterns are rare and unique, as these patterns are typically immutable in natural environment. However, certain groups within the *Clematis* cultivar alter their floral patterns across different seasons, with double flowers blossoming in the spring and single flowers appearing later in the year. This characteristic holds significant ornamental and research value in the study of floral development. The *Clematis* genus occupies a special place within the Ranunculaceae family due to its membership in the apetalous taxa, which enriches the diversity of floral structure in this family. Numerous studies have been conducted to understand the molecular mechanisms of petal loss in wild-type *Clematis* [1–4]. Nevertheless, the seasonal floral-pattern changes within cultivars have received limited attention. Consequently, this study employed the representative cultivar *Clematis*’ Vyvyan Pennell’ to investigate the genetic regulation underlying the phenotype, thereby potentially offering valuable insights into *Clematis* floral development. Similar to other *Clematis* species, ‘Vyvyan Pennell’ has lost petals in the second whorl [5], it retains doubl-flower attributes during spring, which is formed by showy sepals and petaloid organs known as “double perianth.” Conversely, these petaloid organs are not observed in the single-perianth flowers later in the autumn. The origin and development of these additional floral organs are intriguing, and the underlying mechanism of this phenomenon warrants further exploration.

The number, shape, and identity of organs are the main components of floral patterns and show great diversity during floral development. The process of flower formation follows a systematic sequence [6], starting with cell division and culminating in the establishment of morphological features through meristems, triggered by flowering signals. In *Nigella damascena*, the change of floral meristem size seems to result in the alternative of organ number and position [7], *WUSCHEL* (*WUS*) is a vital gene for maintaining cell proliferation in the floral meristem, which an magnify meristem and increase the numbers of floral organs. This gene was negatively regulated by *AGAMOUS* (*AG*), *ULTRAPETAL1*(*ULT1*), *KNUCKLES* (*KNU*), and *SUPERMAN* (*SUP*) to terminate the differentiation of the meristem cells [8–11]. The time and rate of cell proliferation are positively regulated by *AINTEGUMENTA* (*ANT*) [12], or negatively regulated by regulators such as *BIG BROTHER*(*BB*) and *BIG BROTHER-RELATED*(*BBR*) [13]. The *ClAVATA3*(*CLV3*) inhibits floral meristem expansion and reduces the number of stamens and carpels [14]. After the floral meristem is formed, three founder cell identity genes (*DORNRÖSCHEN*, *DORNRÖSCHEN-LIKE*, and *PUCHI*) are combined to initiate the development of floral organs in each whorl [15]. In this process, the floral integrators *FLOWERING LOCUS T* (*FT*) or homologous *HEADING-DATE 3A* (*Hd3a*) in *Oryza sativa* [16]*,* and *SUPPRESSOR OF OVEREXPRESSION OF CONSTANS1* (*SOC1*) activate floral meristems’ identity genes such as *LEAFY* (*LFY*), *APETALA 1*(*AP1*), *UNUSUAL FLORAL ORGANS*(*UFO*) to promote the floral meristem fate [17]. Then the floral organ identity genes directly regulated the formation of the floral structure.

Over three decades dedicating on floral development, “the essential floral organ model” (“the classical ABC model”) has been formulated [18, 19]. This model has facilitated a comprehensive understanding of the genes responsible for the spatio-temporal expression of floral organs. In accordance with this framework, a “typical” four-whorl floral structure, consisting of sepals, petals, stamens, and carpels, is formed through the interplay of A-, B-, and C-class genes. These genes, either individually or in combination, determine the specific identities of floral organs: A, sepals; A and B, petals; B and C, stamens; C, carpels. Additionally, the A-/C-class genes mutually repress each other, thereby restricting their respective expression regions [19]. The loss or overexpression of these floral organ identity genes can result in the conversion of organs and alterations of patterns [17, 20]. To a certain extent, this model is widely conserved among the higher core eudicot flowers, which comprise four typical whorls. For monocot plants, some of them deviate from this model. For instance, in the Liliaceae family, the two outer whorls have the same form of petal-like organs called “Tepals.” In this situation, the expression of the B-class gene expands in whorl 1, which can be elucidated by “the modified ABC model” (“the sliding boundary model”) [17, 21]. In certain lineages of basal angiosperms, such as Nymphaeaceae and Amborellales, the tepals exhibit a gradual transition from sepal-like to petal-like appearance, resulting in an indistinct and variable demarcation between the outer two whorls, referred to as the “fading border model”. In this case, the boundaries between neighboring organs are blurred and uncertain [22]. These phenomenons suggest that plants display different expression patterns of organ identity genes, and even slight changes in expression can impact the diversity of floral structures.

Besides differences in floral development, ambient temperature and endogenous hormones are essential for the regulation of floral structure. In some varieties, such as *Dianthus caryophyllus* ‘Cherie’ and *Cyclamen persicum* ‘Wink Pink II,’ the number of petals changes in response to artificially controlled low- and high-temperatures [23, 24]. Typically, relatively cold temperatures (ranging from 5 to 15 ℃) stimulate more floral organs, which were often originated from proliferating petals or petaloid stamens. Conversely, higher temperatures (ranging from 20 to 26 ℃) can facilitate the normal development of stamens. In the study of double-flowered *Lilium hybrid* ‘Red Twin’ [25], it was observed that the expression levels of *LrtAG1* were reduced at relatively low temperatures, resulting in a narrower expression region and the development of petaloid stamens. Similarly, in *Rosa chinensis*’ Old Blush’, the *APETALA 2* (*AP2*) gene was found to be a significant factor contributing to the formation of petaloid stamens under different temperature conditions [26]. Both stamens and petaloid stamens exhibited considerable thermosensitivity. So, at different temperatures, the critical floral organ identity genes were expressed differently, leading to diverse floral phenotypes, which varied among varieties. The impact of endogenous hormones on flowers is substantial, as evidenced by researches indicating that different hormone concentrations can influence the quantity and arrangement of floral organs. For example, the spatial initiation of floral organs can be affected by the antagonistic relationship between auxins and cytokinins; blocking cytokinin signaling can alter the organization of organs [6], while the application of gibberellic acid (GA3) can stimulate the development of additional floral organs or petals in *Lycopersicon esculentum* and *Dianthus caryophyllus *[27, 28]. Conversely, elevated levels of IAA and ABA appear to be linked to stamen-less phenotypes in tomato mutants [29].

These internal and external factors act as switches in floral development and govern intricate networks. It is imperative to investigate potential regulators that are pivotal in seasonal variation of floral patterns. In this study, morphological, transcriptional and metabolic analyses were performed on the *Clematis* ‘Vyvyan Pennell’ to elucidate the molecular mechanism of floral pattern variation. The findings of this study may offer valuable insights into the exceptional floral development of *Clematis*.

## Results

### Morphological characteristics of the two patterns buds

**Fig. 1:**
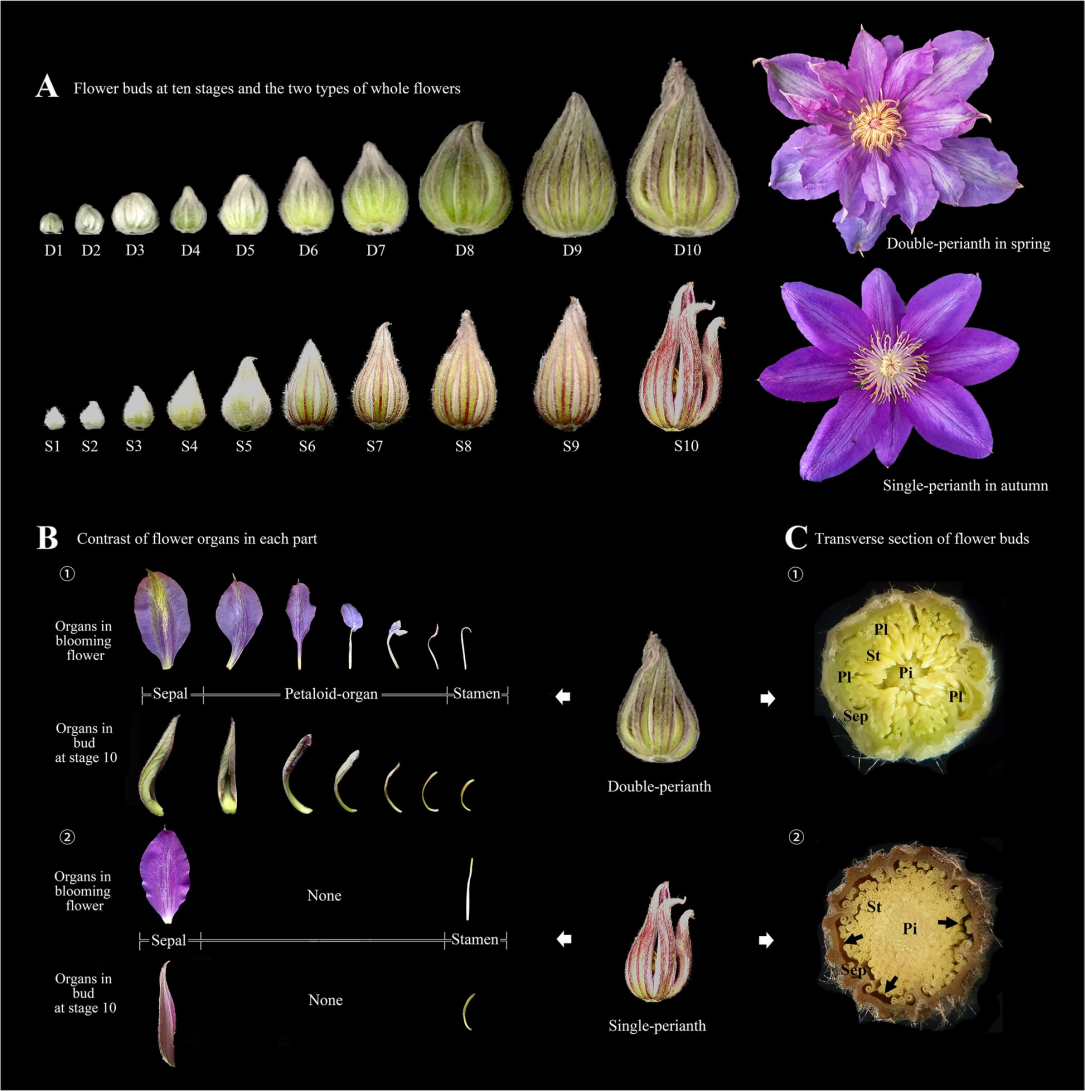
Development stages of two types of buds in *Clematis*’ Vyvyan Pennell’. **A** Morphological observation of floral buds at ten stages and the two types of whole flowers. **B** The contrast of floral organs. The displayed organs positioned vertically correspond to their respective counterparts from the blooming flowers and stage 10th bud separately. ① Organs in the double-perianth flower; ② Organs in the single-perianth bud. **C** Transverse section of floral buds. ① Double perianth; ② Single perianths; Sep: sepal; Pl: petaloid-organ; St: stamen; Pi: pistil; the arrows show the gaps between sepals and stamens

To study the morphological characteristics of the two floral patterns in different seasons, the developmental status was continuously observed. A total of 300 floral buds, 162 double buds in spring and 138 single buds in autumn, were sampled from 50 ‘Vyvyan Pennell’ plants for the purpose of measurement and observation. The growth process was divided into ten stages based on the length and developmental status of the buds (Fig. 1A, Supplementary Fig.S1 and Table.S1). In the initial three stages, both types of floral buds were covered with white trichomes. However, as the buds progressed in development, the trichomes gradually decreased, and the sepals underwent color changes and elongation in subsequent stages, ultimately transforming into petal-like structures after flowering. By arranging the highly mutable floral organs of the outer three whorls (Fig. 1B), the morphology of organs has been shown as a progressive variation from “sepal-like” to “stamen-like”, with trichomes were also observed on the internal petaloid organs. Transverse sections revealed that the inner space of a double perianth was more compact than that of a single bud. In contrast to the multiple layers observed in double bud, a significant gap was observed between the sepals and stamens in single bud (Fig. 1C).

**Fig. 2:**
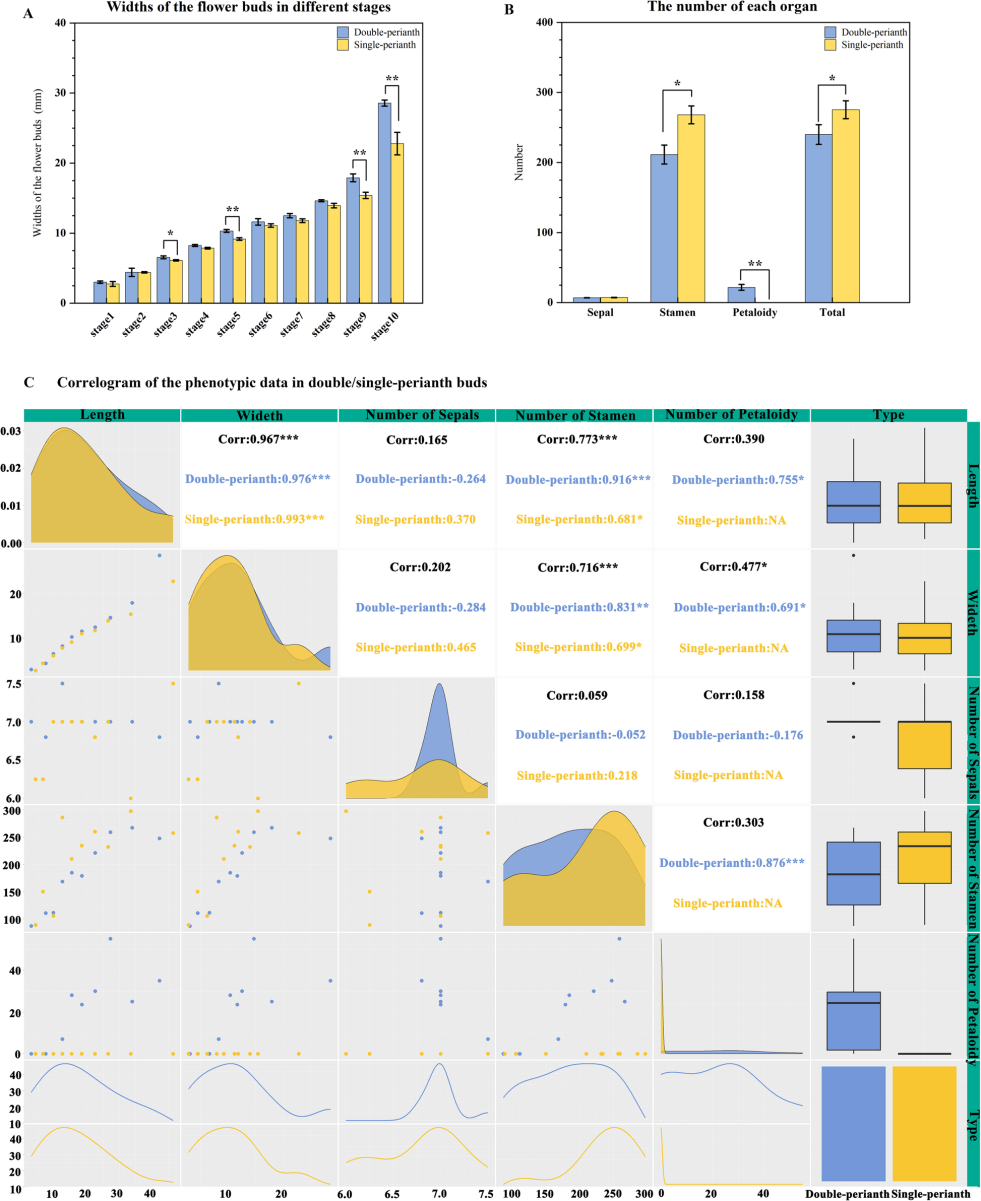
Analysis of the morphological index of two bud types. **A** The diameters of floral buds. **B** The number of floral buds’ organs. **C** Correlogram of the phenotype indexes between the floral buds of two seasons. * represents significant difference, *p* < 0.05; ** represents extremely significant difference, *p* < 0.01; the error bar takes SE value

The diameter of the buds and the quantity of each organ were measured and calculated using a T-test (Fig. 2). The widths exhibited noteworthy disparities at stages 3, 5, 9, and 10 (Fig. 2A), with the 5th, 9th, and 10th stages displaying exceptionally significant differences (*p* < 0.01). The total number of organs exhibited substantial variation, primarily in the quantity of stamens and petaloid organs, while the sepals in double and single flowers remained consistent (Fig. 2B). Correlation analysis of the morphological indices (Fig. 2C) revealed a robust association between the length and width of both bud types. Furthermore, the number of stamens and petaloid organs was positively correlated with bud width. In addition, there was a positive correlation between the number of stamens and petaloid organs in spring double-flower buds. These findings suggest that the petaloid organs in spring flowers may be transformed from stamens, and the development of these additional petal-like structures may be the primary factor contributing to the seasonal variation.

### Observation of the buds and the origin of the petaloidy 

**Fig. 3:**
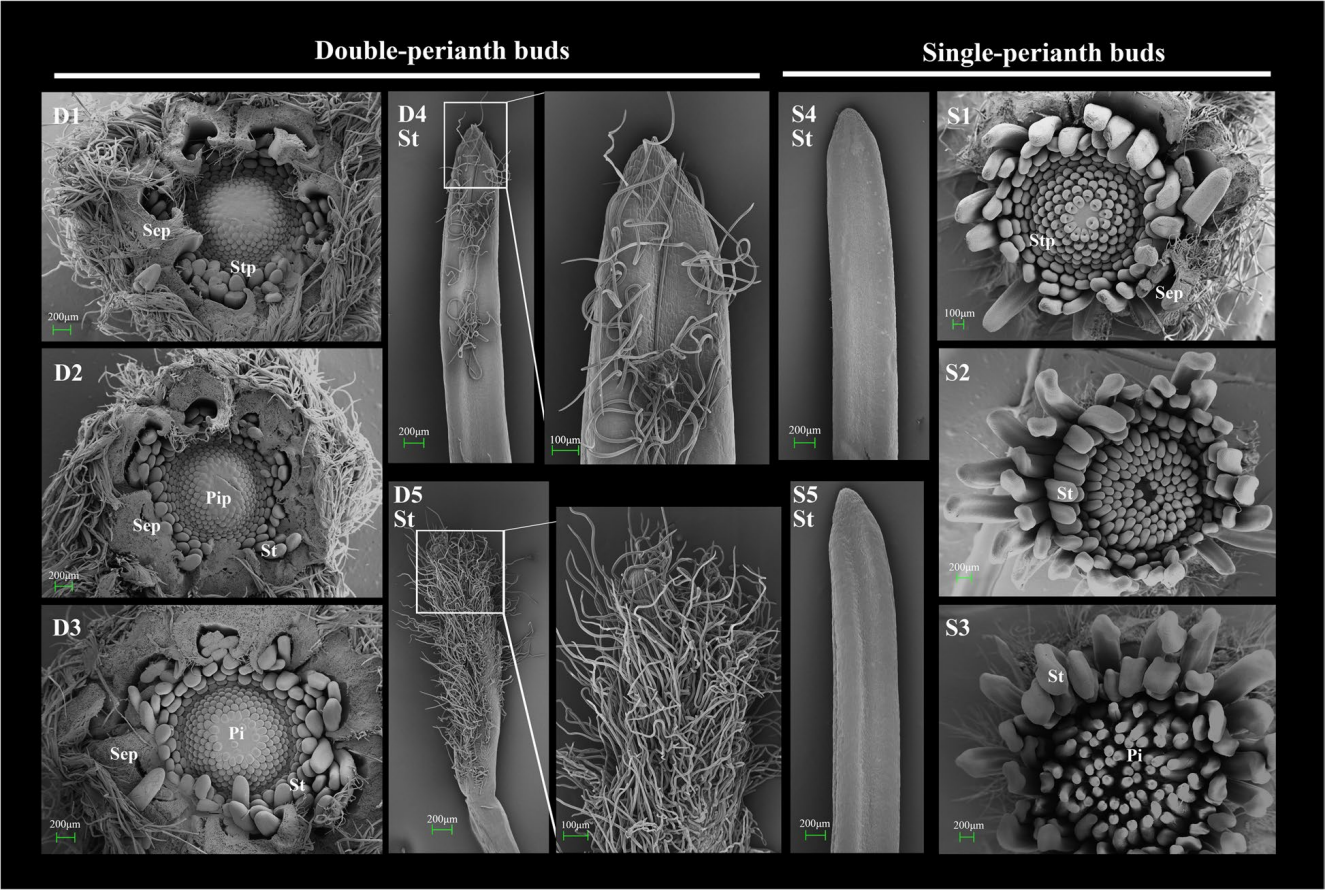
Differences in two seasonal buds and stamens under the scanning electron microscope. D1–D5: stages 1–5 in spring double-perianth buds; S1–S5: stages 1–5 in autumn single-perianth buds; Sep: sepal; Stp: stamen primordium; St: stamen; Pip: pistil primordium; Pi: pistil

To investigate the formation of petaloidy and its timing, we observed the development of two seasonal buds and the outermost stamens using stereomicroscope and SEM (Supplementary Fig.S1 and Fig. 3). Anatomical observations indicated that there were no significant differences between the two types of buds during the initial three stages. However, starting from the 4th stage, the internal structure of double-perianth buds underwent notable changes, diverging completely from that of single buds (Supplementary Fig.S1). Additionally, trichomes were observed on the apex of the outermost stamens (Fig. 3 D4St). The presence of petaloid organs becomes evident in the 5th stage, with the transformation of stamens into petal-like structures and the further growth of trichomes (Fig. 3 D5St). Therefore, this stage can be considered as the critical period for the emergence of petaloidy. By stage 7, the petaloid organs, stamens, and pistils in the double bud had reached full development (Supplementary Fig.S1). Subsequently, in the 9th stage, the sepals underwent petaloid transformation, resulting in significant changes in color and shape on both flanks, thereby increasing their resemblance to petals (Fig. 1A). The observation of each stage indicates that the petaloid organs were not initially present but gradually transformed from stamens during the development of the spring bud. The 5th and 7th stages marked the initiation and completion of stamens’ transformation, respectively, while the 9th stage represented a crucial phase in the petaloid transformation of sepals.

### Transcriptome analysis and functional annotation

Based on these morphological observations, two seasonal floral buds and their organs at the 5th and 7th stages were selected for transcriptome sequencing. A total of 561.48 Gb raw reads were generated from 42 libraries, – 3 biological replicates of 7 samples (double perianth: buds, sepals, petaloid organs, and stamens; single perianth: buds, sepals, stamens) from stage 5 and 7, with high-quality reads accounting for > 90% of the reads after cleaning and filtering. The Q20 value for each sample exceeded 95.36% (Supplementary Table.S3 A), indicating that the sequencing quality met the requirements for subsequent analysis.

A total of 209,056 unigenes were obtained from the de novo assembly of the clean reads with a mean size of 855.41 bp, N50 of 1158bp and an average GC content of 40.36% (Supplementary Table.S3 B). All these unigenes were aligned to the six databases, which are NR, eggNOG, Swiss-Prot, Pfam, GO, and KEGG, to determine their possible functions; 56,521 (27.04%), 51,709 (24.73%), 33,409 (15.98%), 22,778 (10.90%), 17,636 (8.44%), and 16,693 (7.98%) annotated information were detected, respectively. Among the total unigenes, 5826 (2.79%) genes were annotated successfully in the six databases (Supplementary Fig.S2 A). In the NR database, the annotated unigenes with E-values < 1e-30 reached 24,378 (43.13%) (Supplementary Fig.S2 B), and 5549 annotated unigenes shared > 80% similarity (Supplementary Fig.S2 B). Homology analysis showed that 10,038 (17.76%), 9828 (17.39%), and 1640 (2.9%) annotated unigenes shared high homology with *Nelumbo nucifera*, *Vitis vinifera*, and *Fragaria vesca* subsp. Vesca, respectively (Supplementary Fig.S2 B).

In the GO database, 50 items related to biological processes, cellular components, and molecular functions at level 2 were aligned (Supplementary Fig.S2 C) [30]. These unigenes are highly enriched in the “Metabolic process” (8587) and “Cellular process” (8296) of the biological process; the cellular component mainly focused on “Cell” (5704), “Cell part” (5651), and “Membrane” (5271), and the molecular function mainly focused on “Binding” (8643) and “Catalytic activity” (8466). In the KEGG database, 33 items at level 2 were annotated (Supplementary Fig.S2 D) [31–33], among which the highest unigene with 1525 genes was attributed to “Signal transduction,” the other genes were highly enriched in “Translation” (1521), “Carbohydrate metabolism” (1392), “Folding, sorting and degradation” (1073), and “Transport and catabolism” (917).

### Differentially expressed genes between two season phenotypes

**Fig. 4:**
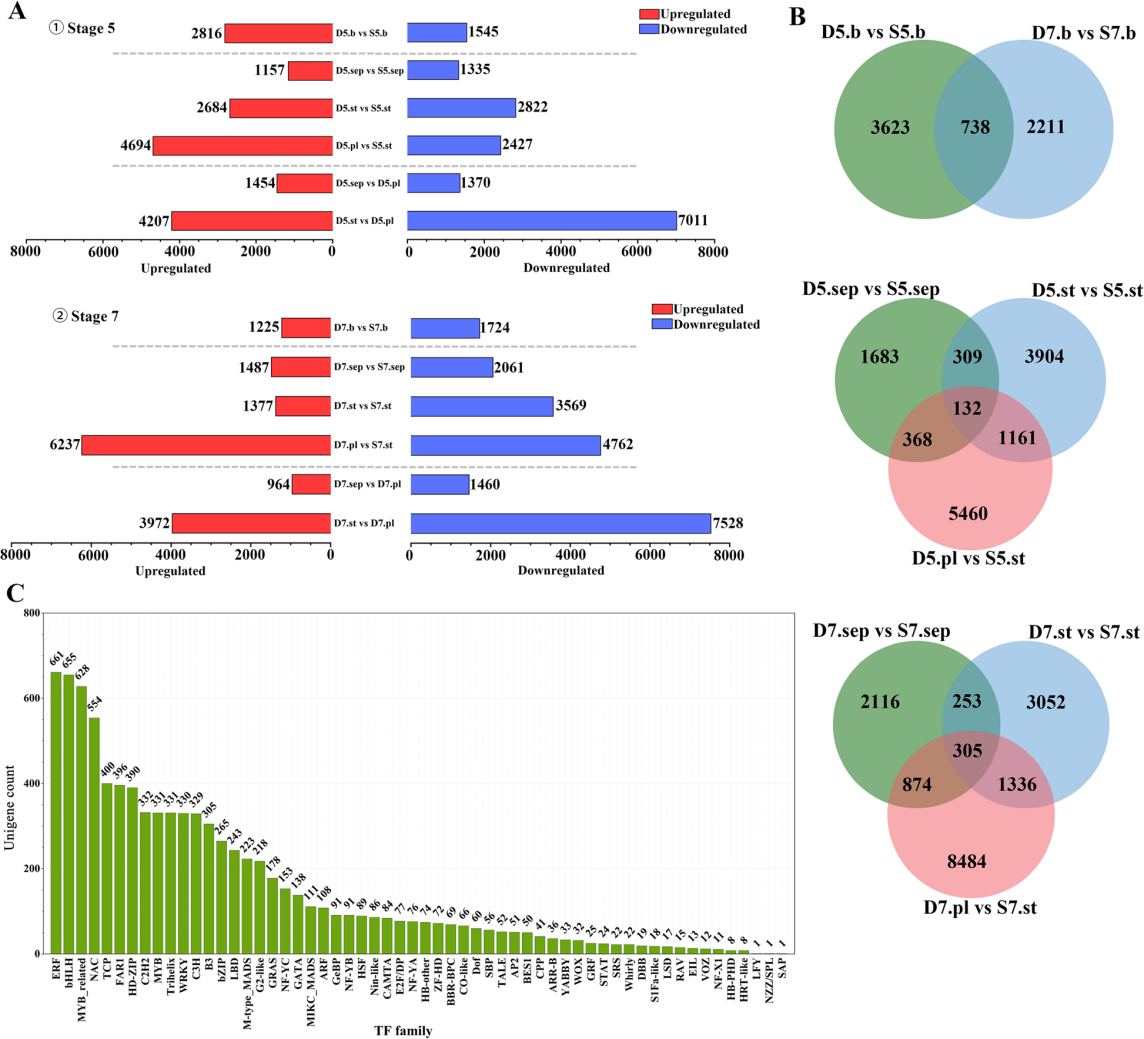
The number of DEGs in different comparison groups and the count of the TF family. **A** Bar chart of the DEGs in each comparative group. ① The DEGs at stage 5; ② the DEGs at stage 7; **B** Venn diagram of different comparison groups; and **C** the count number of genes in different TF families

According to the analysis of the gene expression levels in each sample, 69,888 differentially expressed genes (DEGs)(|log_2_FC|> 1, *p* < 0.05) were screened from the comparative groups. By comparing the number of DEGs between the groups (Fig. 4 A), the most significant difference between the two seasons was identified. Firstly, it was observed that the bud contrast group exhibited a higher number of DEGs at stage 5, a critical period for petaloid organ formation, when compared to stage 7. These DEGs, consisting of 2816 upregulated genes and 1545 downregulated genes, may play a role in the regulation of petaloidy. Secondly, when contrasting the floral organs from different seasons, it was found that the number of DEGs between petaloid organs and stamens (pl vs. st) was the largest. At the 7th stage, there were 6237 upregulated genes and 4762 downregulated genes associated with this contrast. Finally, comparing the differences among organs in spring, the number of DEGs between the stamen and petaloid organs was still the highest. A comparative analysis of common and unique genes among seasonal buds (D5.b vs. S5.b, D7.b vs. S7.b) revealed 738 common genes and 3623 and 2211 unique DEGs (Fig. 4 B). Among the organ comparison groups, stage 7 exhibited a higher number of common DEGs (305) compared to stage 5 (132). The comparison between D7.pl and S7.st showed the highest number of unique differential genes (8484), while the comparison between D5.pl and S5.st had 5460 unique DEGs. It is postulated that there exists significant developmental heterogeneity between petaloid organs and stamens, all these DEGs between petaloid organs and stamens may play a role in the stamen homology transformation. Therefore, we focused on the comparison groups of stamen vs. petaloidy, which were D5.pl vs. S5.st, D5.st vs. D5.pl at stage5, D7.pl vs. S7.st, and D7.st vs. D7.pl at the 7th stage. GO and KEGG enrichment analyses were performed to further analyze the functionality of the DEGs in these comparisons (Supplementary Fig.S3)[30–33]. The GO enrichment showed that the DEGs of different seasons (D5.pl vs. S5.st, D7.pl vs. S7.st) were significantly enriched in GO terms of “catalytic activity,” “membrane,” “intrinsic component of membrane,” and “integral component of membrane.” A similar result was observed in the comparison of D5.st vs. D5.pl and D7.st vs. D7.pl (Supplementary Fig.S3 A)[30]. KEGG enrichment analysis indicated that “phenylpropanoid biosynthesis,” “plant hormone signal transduction,” and “starch and sucrose metabolism” were the three most abundant pathways in the four comparisons. In particular, the pathway related to “plant-pathogen interaction” was significantly enriched in D7.pl vs. S7.st (Supplementary Fig.S3 B)[31–33]. Overall, in the seasonal variation of floral patterns, the functional classification of the DEGs involved metabolite synthesis, transduction and activity, energy metabolism, and membrane changes.

Transcription factors (TF) are essential for plant growth and development. By analyzing the transcript domains, 8782 unigenes from 58 TF families were annotated (Fig. 4 C). Most transcripts were assigned to ERF (661), bHLH (655), MYB-related (628), NAC (554), and TCP (400), which have been proven to play important roles in floral development, hormone responses, and metabolite biosynthesis. In addition, the MADS-box family, including M-type MADS (223) and MIKC MADS (111), has been the focus of research, as this family is a key regulator of floral organ morphogenesis. Of all the genes in the MADS family, 43 were differentially expressed across organs (Supplementary Table.S4), and some were critical candidate genes involved in seasonal variation of floral patterns.

### Critical genes involved in the formation of floral structure

**Fig. 5:**
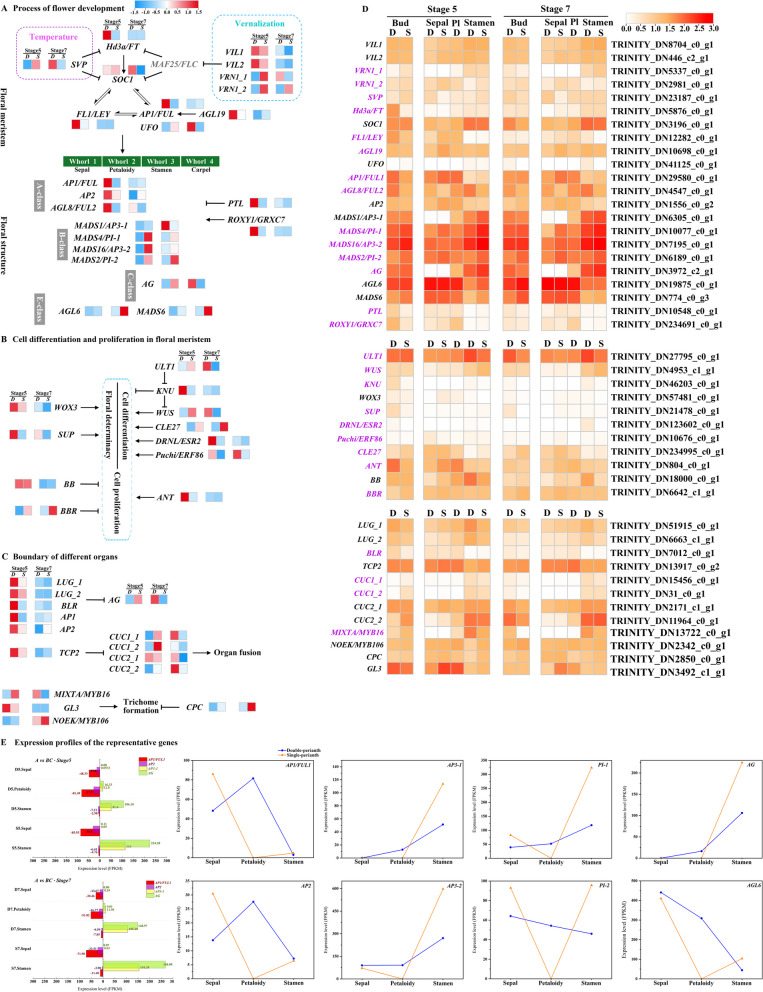
Schematics of flower development, floral meristem cell differentiation and proliferation, and organ boundary-related genes. **A** Genes involved in the process of floral development; **B** Critical genes related to cell differentiation and proliferation in floral meristem; **C** The boundary genes of different whorls of floral organs and the genes related to trichome formation; the heatmap in (A)(B)(C) showed the normalized FPKM transformed values in different seasonal buds at stages 5 and 7. D represents double-patterned buds in spring, S represents single buds in autumn, red denotes high expression, and blue denotes low expression; **D** heatmap showed the log_10_(FPKM + 1) transformed gene expression values in buds, sepals, petaloid organs, and stamens at different stages, the redder in color, the higher the expression. The genes with significantly different expressions between buds at stage 5 were highlighted with purple fonts. **E** Different expression patterns of the A-, B-, and C-class genes which directly regulated floral structure

In order to ascertain the crucial genes linked to variations in floral structure across different seasons, a total of 111 candidate DEGs, encompassing functions related to flowering, floral development, cell differentiation and proliferation within the floral meristem, as well as the boundary of floral organs, were identified based on their significant differential expression. Among these, 48 genes with high expression levels or important functions in the buds and organs were selected (Fig. 5). During the initial phases of floral development, the B3 domain-containing transcription factors *VERNALIZATION 1* (*VRN1*) and *SHORT VEGETATIVE PHASE* (*SVP*) from the MADS-box family exhibit significant differences in expression levels within seasonal buds. These genes have been demonstrated to be involved in the flowering pathways of vernalization and temperature [34, 35]. In addition, we found that the MADS-box protein *AGAMOUS-LIKE 19*, responding to vernalization by short cold periods in an *FLC*-independent manner, was highly expressed in spring buds [36]. This suggests that temperature may be involved in the variation of floral patterns between the two seasons. The homologous gene *HEADING-DATE 3A* (*Hd3a*), akin to *FLOWERING LOCUS T* (*FT*) and serving as an integrator of flowering pathways, displays high expression levels in early-stage spring buds. In contrast, another integrator gene, *SUPPRESSOR OF OVEREXPRESSION OF CONSTANS1*(*SOC1*), exhibits predominant expression in stamens and demonstrates differential expression patterns among buds in later stages. Furthermore, the floral-meristem identity genes *LEAFY*(*LFY*) and *APETALA 1*(*AP1*) were observed to be specifically high expressed in spring buds and petaloid organs (Fig. 5A).

Notably, there were significant differences in the expression levels of A-, B-, and C-class genes between seasonal buds and organs (Fig. 5AD). Specifically, two A-class genes (*AP1/FUL1* and *AGL8*/ *FUL2*), four B-class genes (*AP3-1*, *AP3-2*, *PI-1*, and *PI-2*), and one C-class gene (*AG*), exhibited exceptionally high expression levels. The A-class genes were highly expressed during the early stages of spring bud development. Conversely, the B- and C-class genes predominantly exhibited high expression in autumn single buds, with the exception of *AP3-1*, which also displayed elevated expression in spring buds, particularly at the 7th stage (Fig. 5A). Interestingly, the expression regions of A-class genes expanded to the petaloidy and the third whorl of stamens (Fig. 5E), while the B-class gene *AP3-1* and C-class gene *AG* exhibited limited expression in the outer whorl of sepals and were primarily expressed in stamens. All three classes of genes were expressed in the petaloid organs, which was consistent with the diversification of their morphology from “sepal-like” to “stamen-like.” Previous studies on various species have demonstrated that A- and C-class genes mutually repress each other’s regulatory functions and restrict their own expression regions [37]. In this case, the expression patterns of the A- and C-functional genes were different from those in the classical model, exhibiting a broader range of expression. In terms of the specification of floral organ boundaries, apart from the A- and C-class genes, *LEUNIG* (*LUG*) and *BELLRINGER* (*BLR*) were two well-characterized boundary genes with the ability to confine *AG* expression from the outer two whorls [38, 39]. Notably, only BLR displayed a significant disparity between spring and autumn buds (Fig. 5 C). This observation implies that in spring buds, *AG* was more restricted than the A-class genes. Regarding the lateral boundary of organs within each whorl, it is worth noting that *CUP-SHAPED COTYLEDON 1* (*CUC1*) and *CUP-SHAPED COTYLEDON 2* (*CUC2*) play a significant role, as the absence of their functional activity can lead to the fusion of organs [40]. The expression of these genes, as observed in the spring season (Fig. 5 D), was found to be relatively low, implying the occurrence of organ fusion in double-perianth flowers. These expressions elucidate the reason behind the reduced total number of double-perianth organs compared to that of single perianth.

For floral meristem cell differentiation and proliferation, several genes that regulated meristem size or determined organ identities, such as *KNUCKLES* (*KNU*), *WUSCHEL* (*WUS*), *SUPERMAN* (*SUP*), *AINTEGUMENTA* (*ANT*), exhibited varying expression levels between stage 5 buds (Fig. 5 B). However, their expression levels were relatively low (Fig. 5 D) due to the near-completion of floral primordium differentiation and identity determination at this stage. Furthermore, we observed high expression of *MIXTA/MYB16* in double-perianth stamens, suggesting its potential role in trichome formation on stamen tips.

### Spatio-temporal expression patterns of the floral organ identity genes

**Fig. 6:**
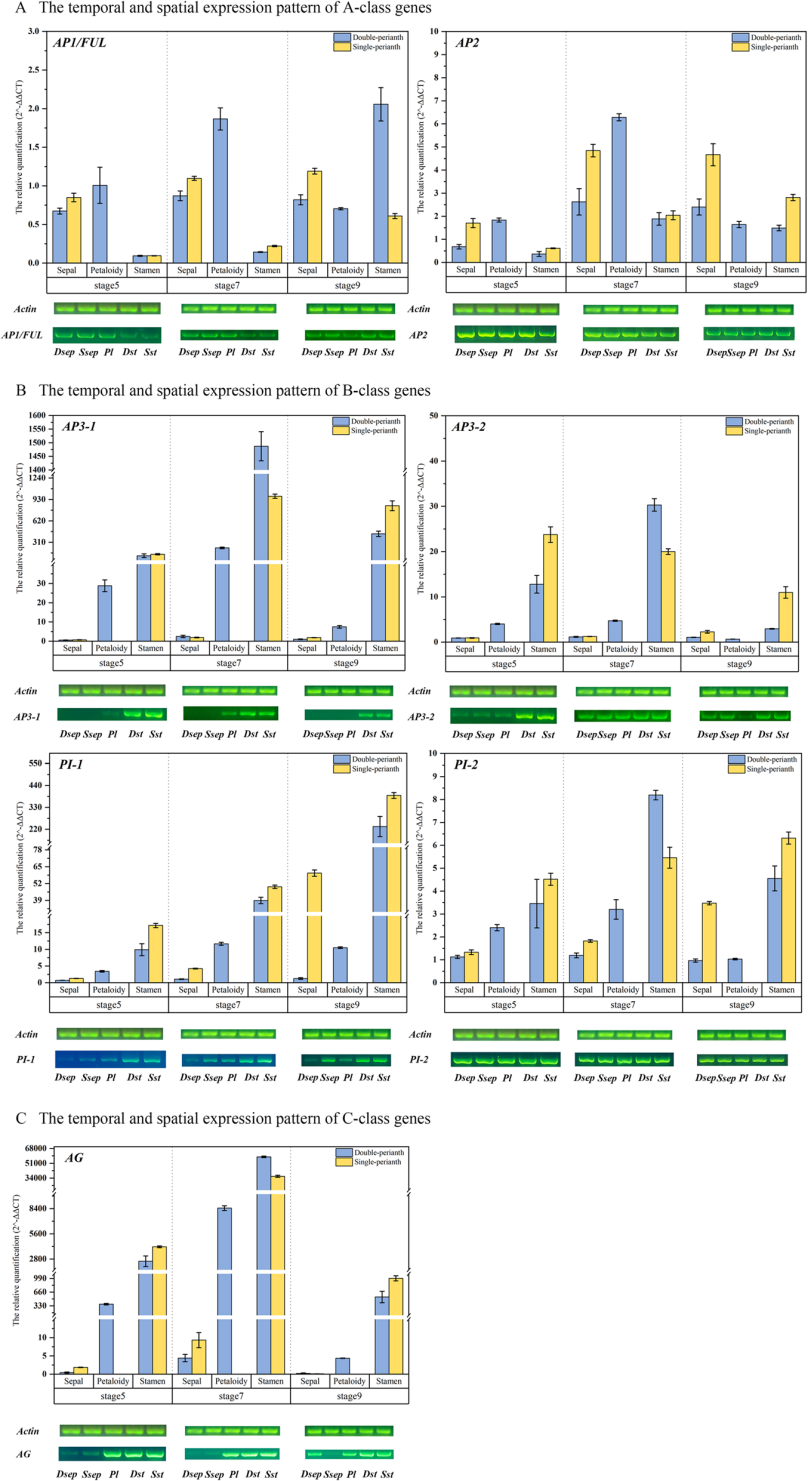
RT-qPCR and RT-PCR validation of the ABC genes. **A** *AP1/FUL* and *AP2* gene expression levels in three organs at the 5th, 7th, and 9th stages; **B** the expression pattern of four B-class genes; **C** the spatio-temporal expression pattern of *AG* genes. The upper chart showed the results of RT-qPCR and the lower images showed the amplified results of RT-PCR; the error bar in the chart takes the SE value

Due to the unique expression patterns of A-, B-, and C-class genes, RT-qPCR and RT-PCR were used to validate the temporal and spatial expression patterns (Fig. 6). The results showed that *AP1/FUL* and *AP2* exhibited similar expression patterns, except that *AP1/FUL* displayed particularly high expression in the stamens of spring flowers at stage 9 (Fig. 6A). This observation was further corroborated by the RT-PCR analysis. It was confirmed that the *AP1/FU*L gene extended its influence to the third whorl of the stamens. On the other hand, the expression patterns of the four B-class genes displayed notable distinctions (Fig. 6B). The results obtained from RT-qPCR and RT-PCR analyses were largely consistent, except for *AP3-1*. Specifically, *AP3-1* exhibited minimal expression in the sepals according to the transcriptome and RT-qPCR results, whereas no gene bands were amplified from the sepals in the RT-PCR analysis, even in the petaloid organs at stage 9. *AP3-1* gene was highly expressed at the 7th stage, suggesting that it may play a role in the formation of the double perianth at a later stage. The findings from the analysis of *AG* gene expression in the sepals exhibited minor discrepancies between the two experiments (Fig. 6C). Generally, the *AG* gene was somewhat expressed in the sepals, proving that the boundary of organ identity genes is unclear and unfixed in seasonal floral-pattern variations. Notably, the *AG* gene exhibited significantly higher expression levels in stamens and petaloid organs, while displaying minimal distinction between double and single flowers. In summary, the expression patterns of all seven genes were largely consistent with the transcriptome sequencing results, which proved that the sequencing data could be used to analyze seasonal floral-pattern variations.

### Variation in phytohormone levels

**Fig. 7:**
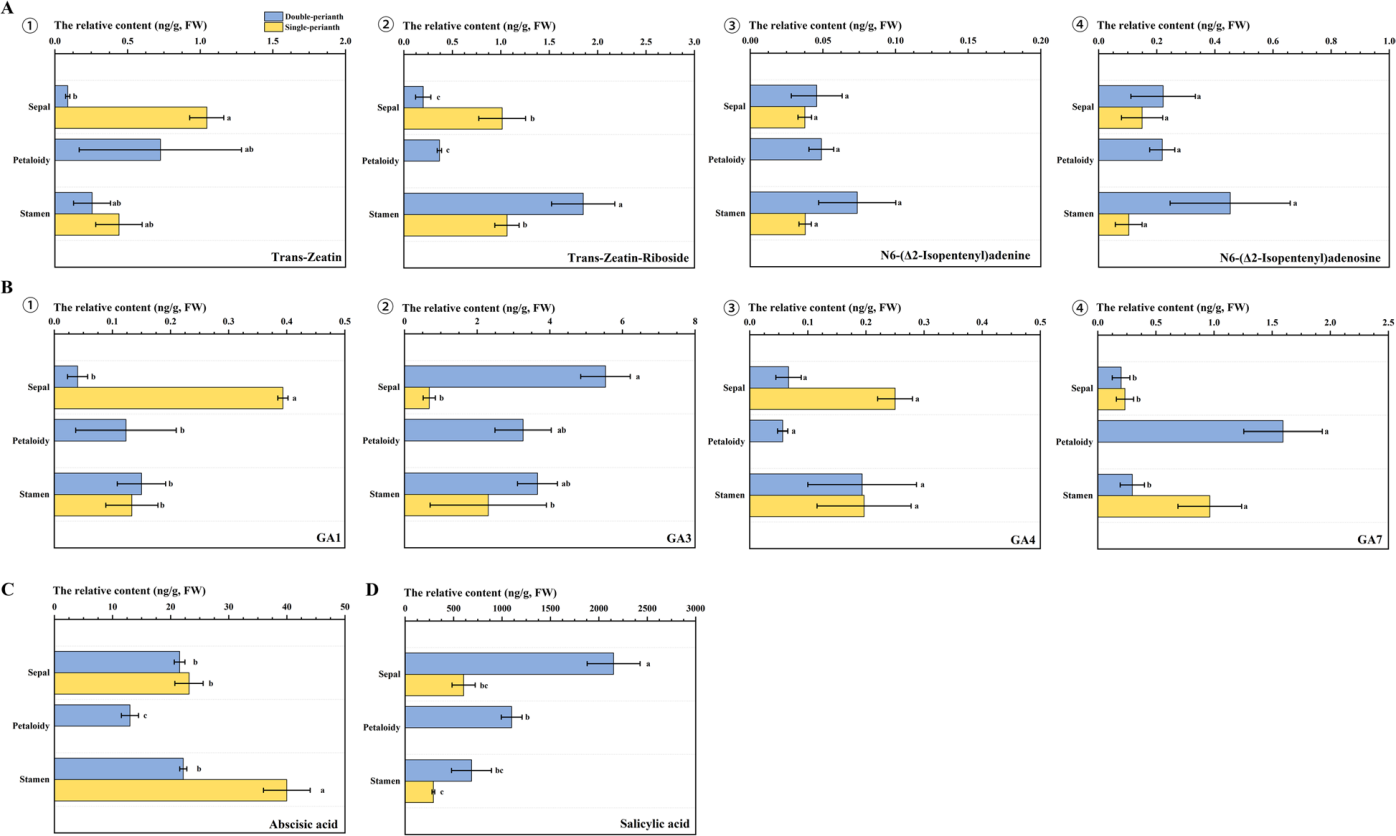
The relative contents of 10 metabolites of 4 phytohormones in the organs of seasonal floral. **A** Relative contents of four cytokinins: ① Trans-zeatin; ② Trans-zeatin riboside; ③ N6-(Δ2-Isopentenyl)adenine; and ④ N6-(Δ2-Isopentenyl)adenosine; **B** Relative contents of four types of gibberellins; ① GA1; ② GA3; ③ GA4; and ④ GA7; **C** The abscisic acid content; **D** The salicylic acid content. The lowercase letters represent a significant difference (*p* < 0.05); the error bar takes the SE value

Plant hormones play important roles in the regulation of floral development and morphogenesis. To identify changes in hormone levels during seasonal floral-pattern changes, 16 metabolites of 8 endogenous hormones were detected. The results showed that brassinolide exhibited the lowest content among the sepal, petaloid organs, and stamens (Supplementary Fig.S4 C). This was followed by N6-(Δ2-Isopentenyl)adenine and N6-(Δ2-Isopentenyl)adenosine of cytokinins (Fig. 7 A③④). Conversely, salicylic acid displayed the highest level of hormones measured in various organs (Fig. 7 D). IAA, ACC, brassinolide, and the three jasmonic acid metabolites did not exhibit significant variations between spring and autumn floral organs. (Supplementary Fig.S4 AD).

Four hormones, namely trans-zeatin, GA1, GA3, and salicylic acid, exhibited notable variations between the sepals of double and single flowers. Specifically, the spring double flower displayed higher levels of GA3 and salicylic acid compared to the autumn single flower, while the remaining two metabolites demonstrated an inverse trend (Fig. 7 A① B①② D). In contrast, GA7 and abscisic acid contents were higher in the stamens of the single perianth than in those of the double perianth (Fig. 7 B④-7C). Notably, the content of trans-zeatin riboside exhibited significant disparities in both sepals and stamens between the two floral patterns, with the highest content in double-perianth stamens (Fig. 7 A②). Based on the aforementioned findings, it is postulated that GA3 and salicylic acid may exert an influence on sepals during the spring season, while trans-zeatin riboside is likely to have an impact on stamens. It is hypothesized that these hormones may play a role in the development of double-perianth pattern.

### Co-expression patterns of genes with seasonal traits and hormone content

**Fig. 8:**
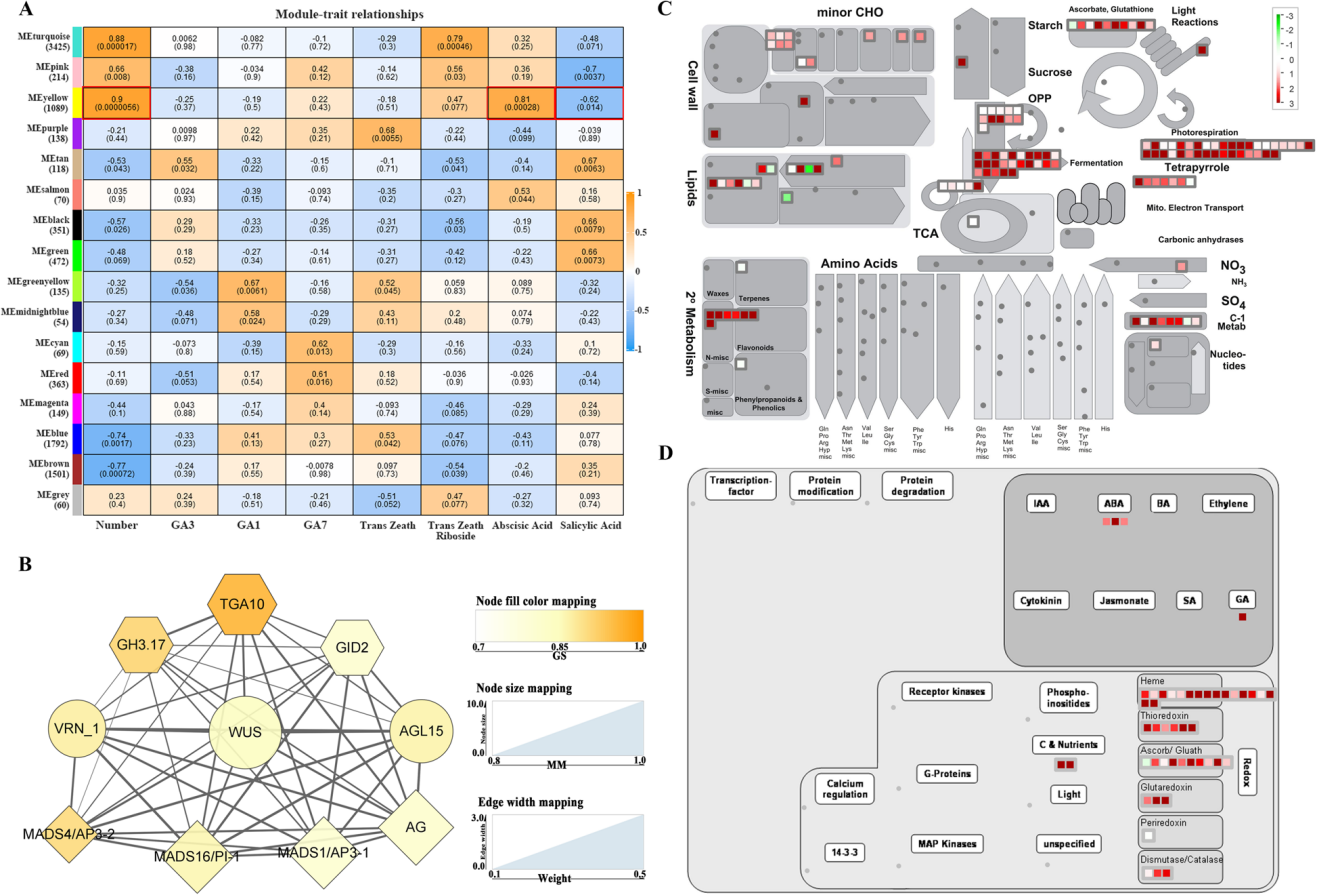
Co-expression network of the genes associated with changes in seasonal floral patterns and hormones. **A** Heatmap of the correlation between gene modules and traits; blue represents negative correlation and orange represents positive correlation; the number in each cell represents correlation coefficient, the number in parentheses represents *p*-value. **B** The co-expression network of 10 hub genes associated with floral development and hormone signal transduction in the yellow module; the diamond nodes represent the floral-identity genes; the hexagonal node represents the hormone-related gene; the node color takes gene significance (GS) value which reflects the correlation between genes and eigengenes phenotype, when the color is darker, the correlation is higher; the node size takes module membership (MM) and reflects the connectivity of genes in this module; the edge width represents the weight value between genes. **C** The metabolism overview of all the annotated genes in the yellow module. **D** The regulation overview of the yellow module genes; red denotes upregulated, and green denotes downregulated

In order to ascertain the hub genes linked to seasonal variation in floral patterns, a co-expressed gene module was derived through the integration of DEGs, seasonal traits, and hormone content by using WGCNA approach. Following the exclusion of genes with insufficient expression from the matrix, representative genes exhibiting similar expression patterns were categorized into 16 distinct modules (Supplementary Fig.S5 B). The gene expression profiles of each module were then examined in relation to the seasonal variations in floral-organ number and hormone content, enabling a module-phenotypic analysis. Notably, the yellow module exhibited the strongest and most significant correlation with the observed traits and content (Fig. 8 A). The eigengenes observed in the yellow module exhibited predominantly upregulated expression in single-perianth stamens (Supplementary Fig.S5 C), suggesting a potential association with the autumn single-perianth phenotype. MapMan annotated all 1089 genes in this module to investigate their functions. The regulation and metabolism overview maps revealed that most of the genes were related to “photorespiration,” “fermentation,” and “heme of redox,” and most of them were upregulated (Fig. 8C-D). Notably, the regulation overview revealed that only ABA and GA were linked to annotated genes, which aligns with the findings that GA7 and abscisic acid levels were higher in single-perianth stamens compared to double perianth (referring to results of hormone content in Fig. 7). Therefore, it is plausible to hypothesize that GA7 and abscisic acid may contribute to the alteration of the autumn single-flower type.

To identify hub genes within the yellow module, we imposed a restriction on the eigengene connectivity value, requiring it to be greater than 0.93, as well as a threshold of 0.90 for the gene significance. As a result, a total of 120 hub genes were retained within this module. Subsequently, through functional annotation, we successfully identified a subnetwork consisting of 10 genes that are associated with flowers and hormones (Fig. 8 B). Among these 10 DEGs, four of them were found to be floral organ identity genes, specifically three B-class genes, *MADS1/AP3-1* (TRINITY_DN6305_c0_g1), *MADS4/PI-1* (TRINITY_DN10077_c0_g1), and *MADS16/AP3-2* (TRINITY_DN7195_c0_g1), and one C-class gene, *AG* (TRINITY_DN3972_c2_g1). It is worth noting that the upregulation of B- and C-class genes may play a crucial role in the single-flower type.

The co-expression network focused on three genes involved in plant hormone signal transduction: *GID2*(TRINITY_DN18105_c0_g1) in the gibberellin signaling pathway, *GH3.17* (TRINITY_DN6388_c0_g1) in the auxin signaling pathway, and *TGA10* (TRINITY_DN8040_c0_g1) in the salicylic acid signaling pathway. The stamens exhibited a higher amount of IAA compared to other organs, however, there was no significant difference observed between spring and autumn flowers (referring to the results of hormone content in Supplementary Fig.S4 A). Therefore, *GH3.17* may not play a critical role in relation to this matter of seasonal variation. Similarly, TGA10 also did not demonstrate significance. Our findings suggest that *GID2* (TRINITY_DN18105_c0_g1) potentially participates in GA7 signaling in stamens, although further experimental validation is necessary. Additionally, the three remaining genes, namely *WUS* (TRINITY_DN4953_c1_g1), *AGL15* (TRINITY_DN30709_c1_g1), and homologous *VRN_1* (TRINITY_DN4971_c0_g1), were identified. These outcomes significantly contribute to the prediction of transcriptional regulation in autumn single-flower stamens.

## Discussion

Flowers, as essential ornamental features in angiosperms, exhibit remarkable diversity in terms of their number, size, color, structure, and fragrance. Among these attributes, the pattern of floral structure has emerged as a subject of significant interest. Generally, floral patterns tend to remain consistent and stable within their natural environment. However, our study reveals that the *Clematis* cultivar ‘Vyvyan Pennell’ exhibits a different morphology in spring and autumn. In order to investigate the mechanisms responsible for the seasonal variation of floral patterns, our study employed morphological observations, transcriptomic analysis, and hormone metabolite detection. Our findings indicate that the expression pattern of floral organ identity genes in *Clematis*’ Vyvyan Pennell’ differs from the established model, suggesting the distinctiveness of this flower formation. Furthermore, we identified 48 candidate genes and 5 hormone metabolites that are potentially associated with the observed seasonal variations in floral patterns. These results contribute to our understanding of floral development in *Clematis* and enhance our knowledge of flower diversity within the Ranunculaceae family.

### Stamens are the key to seasonal variation in floral pattern

According to the continuous morphological and anatomical observations of floral buds and organs in spring and autumn (Fig. 3 and Supplementary.S1), it has been proposed that the pattern change was caused by stamen petaloidy. This transformation has exhibited an influence on the phenotypic indices, including width and organ numbers. Previous investigations on Lily [41] and Cyclamen [42] revealed a consistent trend where an increase in petal count corresponded to a decrease and degeneration of stamens, as they underwent homeotic transformation from stamens into tepals or petals. Similar homeotic conversion of stamens was also observed in our study, which was evident in the alteration of floral organ quantities, with an increase in petaloid organs and a decrease in stamens. Additionally, it was found that single-perianth flowers exhibited a significantly higher number of stamens compared to double-perianth flowers (Fig. 2). This observation substantiates the conversion of stamens into petaloid structures.

Our investigation revealed that the petaloid stamen did not manifest in the initial stages of primordium formation, but rather developed gradually during the growth of the spring bud (Fig. 3 and Supplementary Fig.S1). This transformation aligns with the formation of a typical double flower. *Camellia oleifera* cultivar ‘Huashuo’ (Li et al., 2021) has a stable phenomenon of stamen petaloidy, and this petaloid direction on the top of finger-shaped stamens was not shown in the early primordium but in the middle stage of development. The same process of stamen petaloidy occurs in *Lagerstroemia speciosa* [43] and *Paeonia lactiflora* [44].

For the study of double-flower development, stamen petaloidy or homologous conversion is not an exclusive occurrence. However, within the apetalous taxa of *Clematis*, the identity of the additional structures arising from stamen petaloidy is an intriguing question, as it remains uncertain whether they represent a restored petal or a novel type of organ. Based on the phenotypic features of these organs (Fig. 1B), it can be inferred that these petaloid organs serve as transitional organs between sepals and stamens, rather than being restored petals. Notably, prior to the conversion of the stamen, trichomes were observed to form on the apex of the stamens (Fig. 3). As the number of trichomes increased, the stamens underwent changes. The highly expressed *MIXTA/MYB16* gene, which is associated with trichome formation, was found to be present in the double-perianth stamens (Fig. 5D). Extensive research on various species has demonstrated that trichomes play a developmental role in regulating floral structure [45]. The members of *MIXTA* regulated the conical shape of epidermal cells in *Antirrhinum majus* petals, which can affect petal presentation [46]. Silencing of *GhMYB-MIXTA-Like10* in *Gossypium hirsutum* can suppress petal trichome growth, resulting in abnormal floral buds [47]. In our research, we postulated that the presence of trichomes on the tips of stamens could potentially influence their shape, thereby facilitating the transformation into petaloid structures.

### Mechanisms underlying seasonal variation in floral patterns

Throughout the developmental stages of floral bud, the differentiation and regulation of floral organs are influenced by various internal and external factors, as well as boundary constraints. Minor alterations in gene expression and regulation during this intricate process contribute to the diversity of floral organs [6]. For the construction of floral organs, the particularly related genes were the A-, B-, and C-class genes [19, 37, 48–50]. We successfully identified all the MADS-box DEGs and *AP2-like* genes of the *AP2/ERF* family in our transcriptome. The expression pattern of the A- and C-class genes in *Clematis*’ Vyvyan Pennell’ deviates from the classical model. Previous researches have demonstrated that the A-, B-, and C-class genes maintain their positions to determine organ identity, as the A- and C-function genes mutually inhibit each other, confining their activity to specific regions [19, 37, 50]. However, in our case, the A- and C-class genes exhibit broader expression regions and more flexible patterns, as evidenced in Fig. 5 and Fig. 6.

**Fig. 9:**
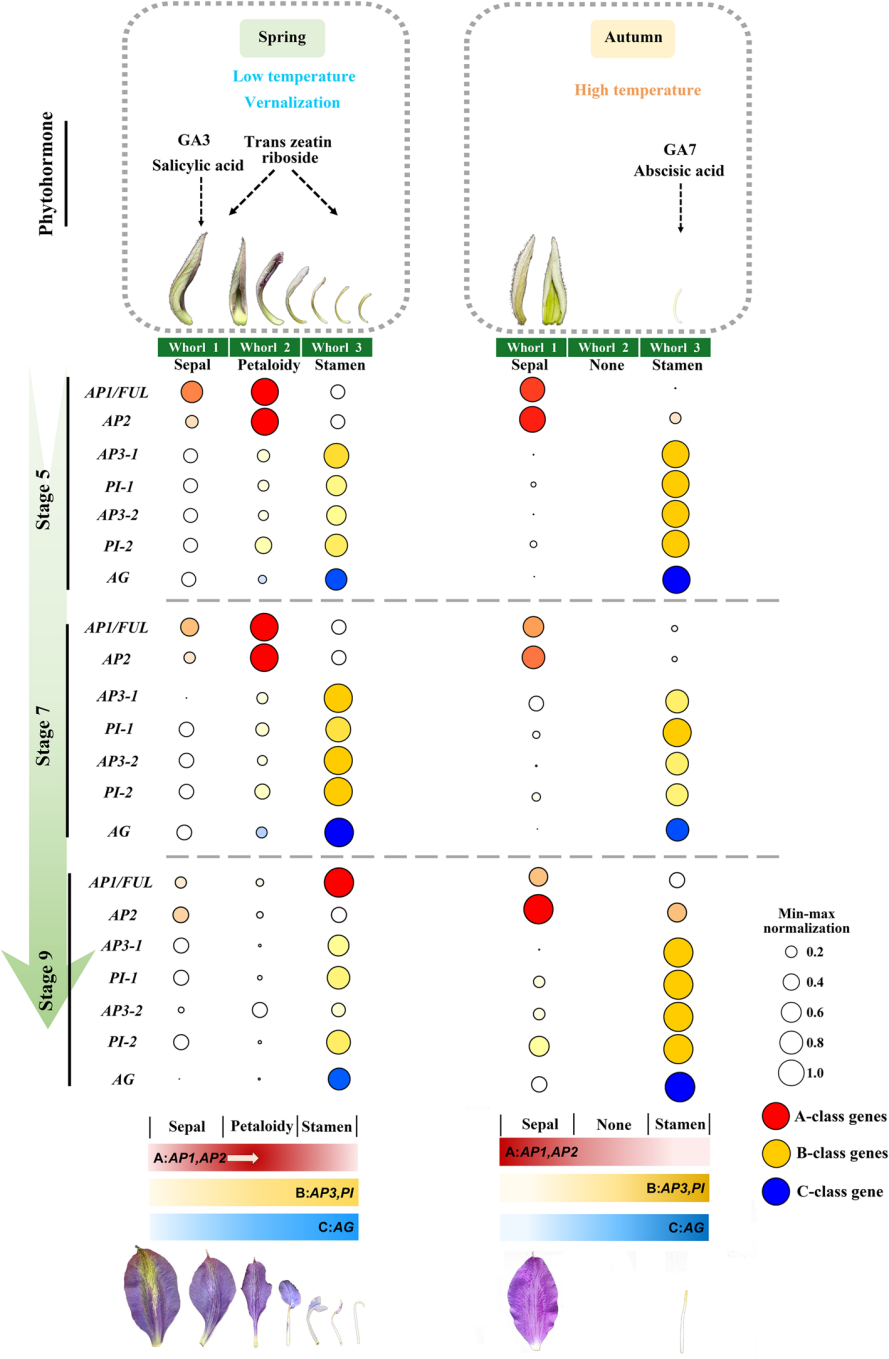
Summary of the mechanism underlying the variations in seasonal floral patterns. The circle size represents the min–max normalized gene expression value in each organ of the three developmental stages. Under the expression, the hypothesized relationship between A-, B- and C-class genes is shown

Generally, in “the classical ABC model,” the formation of a typical double flower is attributed to two pathways: the overexpression of the A-class gene *AP2* [20, 51–54] and the loss of function of the C-class gene *AG* [17, 55–58]. Both approaches ultimately result in the expansion of A-class gene expression and the suppression of C genes, leading to the development of petaloid stamens. However, in this particular study, the A-class gene expanded its expression domain to the inner whorl, while the C-class gene, which also exhibited a broad range of activity. As a result, the mechanism underlying stamen petaloidy in *Clematis* ‘Vyvyan Pennell’ differed from that observed in typical double flowers. Based on the expression levels of the three organ identity genes, it is postulated that A-, B-, and C-class genes collaborate in shaping petaloidy through a modified “fading border model,” while the expression levels of homologous B-class genes dictate organ formation (Fig. 9).

In basal angiosperms, the transition from tepals to stamens occurs due to the gradient expression levels of organ identity genes. The weak expression at the overlapping margins of functional gene-domains leads to the formation of transitional organs with mixed morphological features [59, 60]. The transitional organs are not commonly observed in other phylogenetic branches. *Clematis*, a species belonging to the basal eudicot clade of Ranunculales [22], possessing the transitional organs in the second whorl of double flowers is very novel. By examining the petaloid organs in the double-perianth bud (Fig. 1 B), it was observed that there was a progressive shift in organ morphology from being “sepal-like” to “stamen-like”. This observation implies that the formation of petaloidy is co-regulated by organ identity genes with gradient expression levels. Previous studies have indicated that B-class genes have a more significant impact on the diversity of floral organ morphology, which determines the identity of floral organs, whereas A-class genes primarily determine the location of floral organs [37, 61, 62].

In our study, the border of the outer three whorls was not clear and fixed. The expression of A-class genes exhibited a gradient pattern from the outer to the inner whorls, with a particularly pronounced expression in the second whorl in spring flowers. Conversely, B- and C-class genes displayed an opposite expression pattern. Notably, in autumn flowers, B- and C-class genes exhibited higher expression levels compared to spring flowers, potentially resulting in a specific constraint on the expression of A-genes (Fig. 9). Furthermore, the expression ratio of A- vs. C-genes differed across the outer and inner whorls, suggesting a potential preferential collaboration between B-class genes and the highly expressed genes in the process of organ formation. Therefore, the expression level of B-class genes determines organ identity (Fig. 9).

### Effects of temperature and hormones on floral-patterns

Previous studies have demonstrated that the development of stamens in certain plant species can be influenced by temperature, with the specific effects being contingent upon the varying sensitivities. In a study on *Dianthus caryophyllus* ‘Cherie’, malformed flowers with irregularly developed stamens were induced at 15–20 ℃ [23]. Similarly, short-term high (35 ℃) or low (2 ℃) temperatures may cause high malformation rates of flowers in strawberry cultivars ‘Maehyang’ and ‘Seolhyang’ [63]. While in some other horticultural varieties, such as *Lilium hybrid* ‘Red twin’ [25], and *Rosa chinensis* ‘Old blush’ [26], the effect of temperature creates unique ornamental values of double-flower traits. Until now, the precise mechanism underlying the regulation of floral pattern changes by temperature remains elusive.

Existing studies primarily concentrate on the different expressions of organ identity genes in response to varying temperatures. In the case of the typical flower *Rose chinensis*, it has been observed that lower temperatures (16 ℃:6 ℃) result in an increased production of petals from stamens, potentially linked to the upregulation of *RcAP2* expression. This upregulation may be attributed to the presence of three heat shock elements and one low-temperature response element on the gene promoter [26]. In the *Lilium hybrid* ‘Red twin’, *LrtAG1* of the C-class gene might be correlated with the petaloidy of stamens, as it showed a higher reduction ratio when the floral buds were transferred to a low temperature [25].

In our study, the *Clematis* ‘Vyvyan Pennell’ bloomed double- and single-perianth flowers separately in spring and autumn, which may be associated with different temperatures in the two seasons. In spring, low temperatures (2.2℃) may lead to the downregulation of B- and C-class genes, while A-class genes were highly expressed and localized in the second whorl. Conversely, relatively high temperatures (27.5℃) in autumn led to a reversal in the expression levels of these genes. In addition, we found that *VRN1*, *SVP*, and *AGL19* were differentially expressed in the spring and autumn floral buds. To the best of our knowledge, these three genes are associated with vernalization or low-temperature-induced flowering. Specifically, *AGL19* can function in the *FLC*-independent vernalization pathway and does not need *SOC1* to function, and elevated *AGL19* levels induced by cold temperature can activate *LFY* and *AP1* [34–36, 64, 65]. Although no research has shown that flowering-inducing genes are involved in floral pattern construction at low temperatures, we speculate that they might play a role in the upstream network of regulation. The whole regulatory mechanism of temperature affecting floral morphology require further investigation.

Phytohormones are actively involved in the biological processes of floral development and organ formation. According to advances in understanding hormone function on floral development, we have an overview of their influences on different organs. In initiation and organogenesis, cytokinin, gibberellin, and auxin play a major role in regulating floral meristem size. The petal development is affected by gibberellins, auxin, and jasmonic acid. For stamen development, almost all hormones have been involved in. In the development of gynoecium, auxin is the predominantly regulator [66, 67]. Furthermore, certain hormones also exert control over the number of floral organs. In Carnations, low temperatures promote secondary growth centers, which can increase the number of petals. Similarly, this phenotype can also be promoted using GA3 or IAA at the tip of the shoot [27]. Sawhney (1983) showed that GA3 could induce an increasing number of sepals, petals, and stamens in *Lycopersicon esculentum*, and implied that low-temperature regimes (18 ℃:15 ℃) induced significantly greater numbers of floral organs through increased levels of endogenous gibberellins [28]. An increase in the number of all floral organs in *Arabidopsis* flowers was observed by applying benzylaminopurine (exogenous cytokinin) at different developmental stages [68].

With the in-depth research, the regulations of floral organs were found based on the transduction and feedback between hormones and genetic information. For instance, as above mentioned, the meristem size is determined by cytokinin, gibberellin, and auxin, by *KNOX* genes the gibberellin levels are maintained [69]. As for cytokinin, it can positively regulate the meristem size through feedback pathways of *WUS* and *CLV *[70]. In addition, Floral organ shape is influenced by GA feedback loops involving floral organ identity genes *AG *[71]. It is obviously that there are complex interconnected webs among all the hormones. Many genes controlling floral development or organ identity have been proved also involving in the metabolism regulation of hormones [66]. It is gratifying that the molecular mechanism underlying floral development and hormone pathways has been well studies. However, there are unresolved issues that warrant attention. Specifically, understanding how environmental factors and hormones interact to impact floral diversity remains a crucial objective. Environmental factors such as temperature can affect the ratio and concentration of these hormones, which can further influence the development of floral organs [72].

In our study, it was observed that the sepals of double flowers exhibited a greater amount of GA3 compared to single flowers, which aligns with findings from previous research. Furthermore, the spring floral organs displayed significant levels of salicylic acid and trans-zeatin riboside, alongside GA3. Conversely, the stamens of single-perianth flowers exhibited elevated levels of GA7 and abscisic acid in comparison to double perianth. These findings suggest that gibberellin plays a crucial role in the development of sepals and stamens, although the specific metabolites may differ across various organs. Considering the different concentrations of these hormones between two seasons, we speculated that GA3, salicylic acid, and trans-zeatin riboside may produce at least part of its effect on the formation of the double perianth, while GA7 and abscisic acid may influence the formation of single flowers. To further investigate these hypotheses, future experimental studies focusing on exogenous hormone application and associated gene functions are warranted.

## Materials and methods

### Plant materials and treatment for observation

Buds and floral organs of different seasons were sampled from three-year-old *Clematis*’ Vyvyan Pennell’ at Southwest Forestry University (102°45′27″E, 25°3′50″N), Kunming, Yunnan Province. The average day/night temperature (day, 06:00–18:00h / night, 18:00–06:00) is recorded as 14.02/10.07℃ in the spring. The temperature varies between 2.22℃ and 20.47℃, with nearly 10 days experiencing temperatures below 5℃. In the autumn, the average day/night temperature is measured at 21.5℃/18.7℃, and the temperature ranges from 14.4℃ to 27.6℃. (These data sourced from the Energy Data Platform) From spring to autumn (April to September 2021), every five days, similar-sized buds were separately collected from 50 plants of the same cultivar ‘Vyvyan Pennell’ for morphological observation (Fig. 1). There were 162 double buds and 138 single buds have been measured the lengths and diameters by using a vernier caliper. The number of each organ was then calculated; the difference in the mean was analyzed by a T-test at 0.05 and 0.01, respectively, using SPSS 21.0. A bar chart was drawn using Origin 2021 and the correlogram was illustrated using the GGally package in R (Fig. 2). The samples for anatomical observation were fixed in 50% FAA and observed under a stereomicroscope (OKA Optical Instrument, China) (Supplementary Fig.S1) and scanning electron microscope (SEM; Hitachi S-4800) (Fig. 3). For SEM observations, the samples, stripped of the outer sepals, were dehydrated using a series of alcohol solutions ranging from 70 to 100%, dried in a Hitachi HCP-2 CO2 critical point dryer, and coated with gold–palladium after drying. The images were acquired using a Hitachi S-4800 microscope. Based on the lengths and developmental status of the buds (Supplementary Table.S1), 10 stages were identified. Two critical stages of petaloid organ formation were identified to collect materials for transcriptome sequencing (Table 1). Three biological replicates were collected and the collected samples were quickly frozen in liquid nitrogen and stored at -80 ℃.

**Table 1:** Sampling period and sample labels

Stages	Double-perianth in spring				Stages	Single-perianth in autumn		
	**Bud**	**Sepal**	**Stamen**	**Petaloid-organs**		**Bud**	**Sepal**	**Stamen**
Stage 5	D5.b-1 D5.b-2 D5.b-3	D5.sep-1 D5.sep-2 D5.sep-3	D5.st-1 D5.st-2 D5.st-3	D5.pl-1 D5.pl-2 D5.pl-3	Stage 5	S5.b-1 S5.b-2 S5.b-3	S5.sep-1 S5.sep-2 S5.sep-3	S5.st-1 S5.st-2 S5.st-3
Stage 7	D7.b-1 D7.b-2 D7.b-3	D7.sep-1 D7.sep-2 D7.sep-3	D7.st-1 D7.st-2 D7.st-3	D7.pl-1 D7.pl-2 D7.pl-3	Stage 7	S7.b-1 S7.b-2 S7.b-3	S7.sep-1 S7.sep-2 S7.sep-3	S7.st-1 S7.st-2 S7.st-3

### Transcriptome sequencing

Total RNA was isolated from 42 flower samples using TRIzol Reagent (Invitrogen Life Technologies, Waltham, MA, USA). The integrity, purity, and concentration of RNA were determined using an Agilent 2100 Bioanalyzer (Agilent Technologies, Palo Alto, CA, USA), 1% agarose gel electrophoresis, and a NanoDrop 2000 (Thermo Fisher Scientific, Waltham, MA, USA). Each sample was standardized to have an integrity score > 7.5 for library construction. mRNA was purified from the total RNA using poly T oligo-attached magnetic beads and broken into fragments by divalent cations at elevated temperatures in an Illumina proprietary fragmentation buffer. Random oligonucleotides and Super Script II were used to synthesize the first-strand cDNA and the second-strand cDNA was subsequently synthesized by DNA Polymerase I and RNase H. Purified cDNA fragments were sequenced on an Illumina NovaSeq 6000 platform [73] by BioNovoGene Biotechnology Co. (Chengdu, China).

### De novo transcriptome assembly, gene annotation, and differential gene expression

Raw reads with adapters, low quality (Q value < 20), and high N rate (≥ 10%) were removed using Cutadapt 1.16 [74]. The remaining high-quality clean reads were assembled into a set of unigenes using Trinity 2.5.1. Since there is no published genome sequence of *Clematis L.*, the most complete set of unigenes was used as a reference for this study. To explore the potential function of the unigenes, all sequences were aligned to protein databases, including NCBI non-redundant protein (NR; https://ftp.ncbi.nlm.nih.gov/blast/db/FASTA/), Swiss-Prot (http://www.ebi.ac.uk/swissprot/), Pfam (http://pfam.xfam.org/) [75], and Evolutionary Genealogy of Genes: Non-supervised Orthologous Groups (eggNOG; http://eggnog.embl.de/version_3.0/) [76], using Diamond 0.9.25.126; the unigenes were then annotated to the Gene Ontology [30] (GO; http://www.geneontology.org/) database using Blast2go 2.5.0. In addition, using the KOBAS 3.0 software [77], the unigenes were imported into the Kyoto Encyclopedia of Genes and Genomes (KEGG; http://www.genome.jp/kegg) database to analyze the significant enrichment of pathways (Supplementary Figs.S2 and S3) [31–33].

According to the numbers of clean reads mapped to the reference unigenes in each library, the gene expression was quantified by RSEM 1.2.15; the expression level of each gene was calculated and normalized to fragments per kilobase per million reads (FPKM). Significant differentially expressed genes (DEGs) between two samples were identified at |log2Foldchange|> 1 and *P*-value < 0.05, using DEseq 1.32.0. The selected genes were predicted using Transdecoder software. The Upset graph of statistics in all six databases was drawn using the UpsetR package of R software (https://www.r-project.org/), and the heatmap of the expression patterns of these DEGs was drawn using TBtools V1.117 [78].

### Gene co-expression network analysis

The WGCNA package in R was used to construct a co-expression network. The FPKM values of the expression matrix of DEGs were imported into the software. After filtering out genes with 30% expression lower than 1, 15,008 genes remained for analysis. The soft threshold was determined based on the principle of a scale-free network, and the power for subsequent analysis was taken as R^2^ = 0.85 (Supplementary Fig.S5 A). The FPKM of all the DEGs was analyzed and hierarchically clustered. The clustering tree was divided into correlated modules by a dynamic tree cut with minimum module size = 30 and cut height = 0.25; each co-expression module containing genes with similar expression patterns is represented in different colors (Supplementary Fig.S5 B). The co-expression modules were highly correlated with phenotypic traits, and the phytohormone content was obtained through module-trait analysis. In these co-expression modules, gene significance (GS) > 0.90 and module membership (MM) > 0.90 were selected as candidates. Ten genes associated with floral development were constructed for a gene interaction network, using a weight value > 0.1 to draw the network using Cytoscape 3.8.0 software [79]. Pathway enrichment of the DEGs with high module-trait connectivity was annotated by Mercator4 [80], using MapMan 3.6.0RC1 to visualize the annotation map (Fig. 8).

### RT-qPCR and RT-PCR analyses

ABC gene expression patterns were investigated using quantitative real-time PCR (RT-qPCR) and RT-PCR. The housekeeping gene *ACTIN* was set as an internal control to standardize the relative quantity of validated genes (Fig. 6). RT-qPCR was performed using QuantStudio (Thermo Fisher Scientific) with Hieff qPCR SYBR Green Master Mix Reagent (Yeasen Biotech Co., Shanghai, China). The thermal cycling conditions included an initial denaturation at 95 ℃ for 5 min, followed by 40 cycles of denaturation at 95 ℃ for 10 s, and annealing/extension at 60 ℃ for 30 s. Three biological replicates of each gene were calculated according to the 2^−ΔΔCt^ method. In RT-PCR experiments, the amount of template was carefully adjusted to match that of *ACTIN*. PCR reactions for all genes were performed with 30 cycles except AP3-2 and PI-2, which required 25 cycles at 94 ℃ (30 s), 60 ℃ (60 s), and 72 ℃ (45 s) using 2 × Hieff® PCR Master Mix (Yeasen, Shanghai, China). Products were fractionated on a 1.5% agarose gel for analysis. Primers were designed using the Primer Premier 5.0 software and synthesized by Tsingke Biotechnology Co., Ltd. (Beijing, China) (Supplementary Table.S2).

### Assay and analysis of endogenous hormones

Floral organs were collected during the two seasons to extract their metabolites, and 16 hormone metabolites involving all 8 phytohormones were detected (Fig. 7 and Supplementary Fig.S4). Freeze-dried samples were added to 50% ACN extract solvent, subjected to ultrasonic treatment for 3 min, followed by extraction at 4 ℃ for 30 min, centrifugation at 12,000 rpm, and subsequent filtering. Next, the solutions were filtered through the RP-SPE column, which finally dissolved in 200 μL 30% ACN. The metabolites were analyzed using a UPLC system and Q Exactive (Thermo Fisher Scientific, USA). The chromatographic column was Waters HSS T3 (50 × 2.1 mm, 2.7 μm). The mobile phases A and B were 0.1% acetic acid in ultrapure water and acetonitrile, respectively. The flow rate was 0.3 mL /min and the column temperature was 40 ℃. The elution gradient was set as follows: 0 min, 90% A/10% B; 1 min, 90% A/10% B; 7 min, 90% A/10% B; 7.1 min, 10% A/90% B; 9 min, 10% A/90% B. The external standard method was used for quantification, and sample quantitative analysis was performed based on the calibration curves of the standards.

### Supplementary Information

**Additional file 1: Supplementary Fig. S1. **Anatomical observation and development status in each stage. **Supplementary Table.S1.** Stages division and the description of the buds’ development status.

**Additional file 2. Supplementary Fig. S2. **Functional annotation of unigenes in *Clematis* ‘Vyvyan Pennell’.

**Additional file 3: Supplementary Fig. S3. **GO and KEGG enrichment of the DEGs in compared group between stamens and petaloidy.

**Additional file 4: Supplementary Fig. S4. **The content of plant hormone with no significant difference between seasonal organs in *Clematis* ‘Vyvyan Pennell’.

**Additional file 5: Supplementary Fig. S5. **Soft threshold value, modules, eigengenes expression pattern in yellow of  WGCNA.

**Additional file 6: Supplementary Table. S2 **Primers of floral-organ identity genes.

**Additional file 7: Supplementary Table.S3. **Quality control and summary of the transcriptome sequence.

**Additional file 8: Supplementary Table.S4. **The Log_2_(Foldchange) of 43 DEGs in MADS family.

## Data Availability

The datasets analysed during this study are available in the National Center for Biotechnology Information (NCBI) repository, the accession number is PRJNA979343; the other data are included within the article and its additional files.
