# Supplementary material for: Seasonal variation of two floral patterns in Clematis ‘Vyvyan Pennell’ and its underlying mechanism
Source: BMC Plant Biol. 2024 Jan 2;24:22. doi: 10.1186/s12870-023-04696-9 (PMC10759560; doi:10.1186/s12870-023-04696-9)
Supplement: Supplementary file 1 — Additional file 1: Supplementary Fig. S1. Anatomical observation and development status in each stage. Supplementary Table.S1. Stages division and the description of the buds’ development status. [file 12870_2023_4696_MOESM1_ESM.pdf]

Supplementary Fig.S1 Anatomical observation and development status in each stage

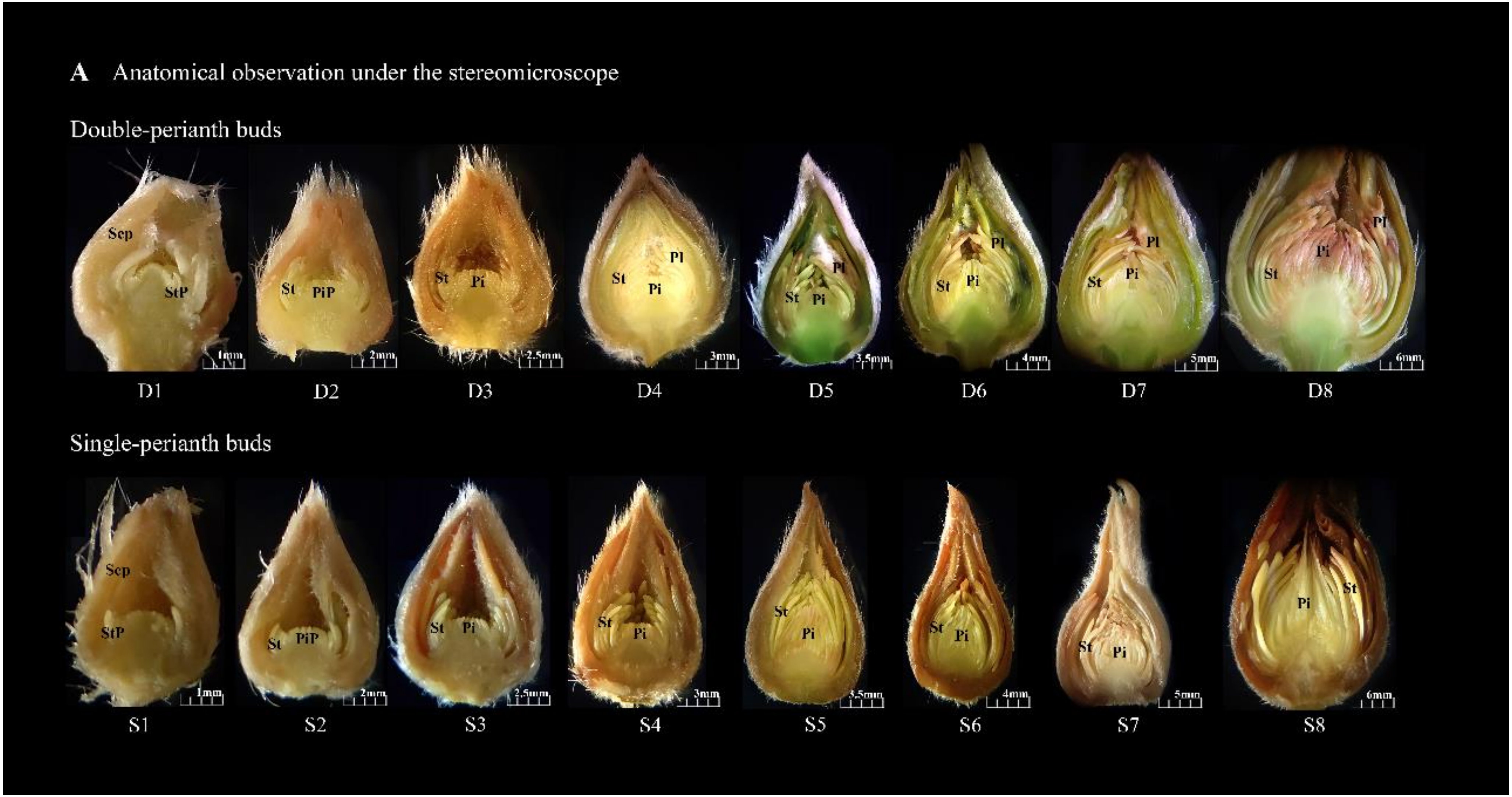

Supplementary Table.S1 Stages division and the description of the buds' development status

**Stages of division**	**Lengths**	**The status of flower buds**		**Figure S1**
		**Double-perianth**	**Single-perianth**	
Stage 1	<5mm	From sepal primordium to sepals completely developed, stamen primordium emerged;	From sepal primordium to sepals completely developed, stamen primordium emerged;	D1: Sep, StP<br>S1: Sep, StP
Stage 2	5-7.99mm	Stamens elongated and pistil primordium emerged;	Stamens elongated and pistil primordium emerged;	D2: St, PiP<br>S2: St, PiP
Stage 3	8-10.99mm	Stamens increased inwards and continued elongation; pistils elongated;	Stamens increased inwards and continued elongation; pistils elongated;	D3: St, Pi<br>S3: St, Pi
Stage 4	11-13.99mm	Stamens continued elongation and *began to change with some white trichomes* on them, pistils continued differentiation and elongation;	Stamens continued elongation, pistils continued differentiation and elongation;	D4: Pl, St, Pi<br>S4: St, Pi
Stage 5	14-16.99mm	*Petaloid organs continued forming*, stamens and pistils continued elongation;	Stamens and pistils completed development;	D5: Pl, St, Pi<br>S5: St, Pi
Stage 6	17-19.99mm	*Petaloid organs continued forming and development*, stamens and pistils continued elongation;	**S**epals started to change color;	D6: Pl, St, Pi<br>S6: St, Pi<br>S6
Stage 7	20-24.99mm	*Petaloid organs completed formation*, stamens and pistils also completed development;	Sepals constantly changed color and shape;	D7: Pl, St, Pi<br>S7: St, Pi<br>S7
Stage 8	25-29.99mm	**S**epals started to change color;	Sepals constantly changed color and shape;	D8<br>S8
Stage 9	30-39.99mm	Sepals were undergoing petaloid;	Sepals were undergoing petaloid;	
Stage 10	>40mm	All floral organs were fully developed, ready to bloom;	Ready to bloom;	

Sep: sepal; StP: stamen primordium; St: stamen; PiP: pistil primordium; Pi: pistil; Pl: petaloid-organ
