# Supplementary material for: Seasonal variation of two floral patterns in Clematis ‘Vyvyan Pennell’ and its underlying mechanism
Source: BMC Plant Biol. 2024 Jan 2;24:22. doi: 10.1186/s12870-023-04696-9 (PMC10759560; doi:10.1186/s12870-023-04696-9)

Supplementary Fig.S2 Functional annotation of unigenes in *Clematis* ‘Vyvyan Pennell’

(A) The upset and venn graph of annotation results in 6 databases

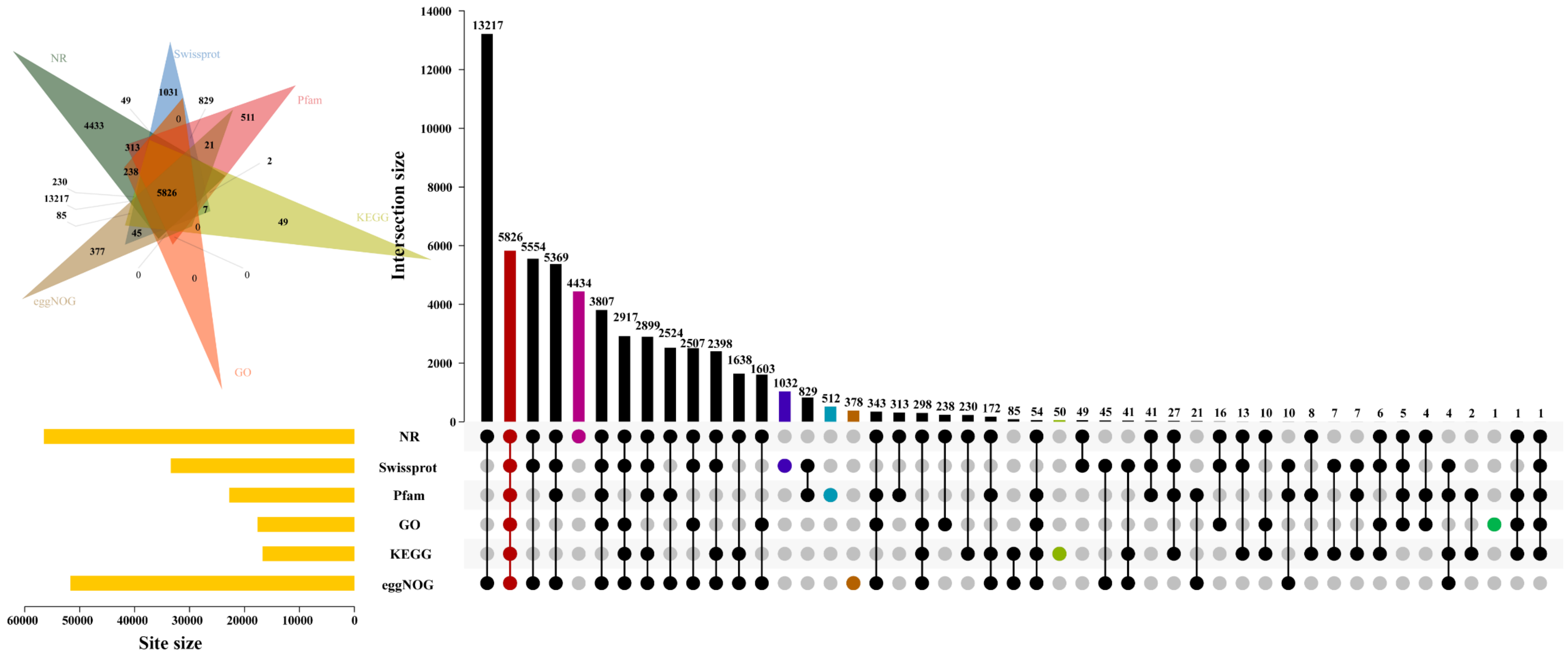

(B) Distribution of the NR E-value, the NR identity and the NR species

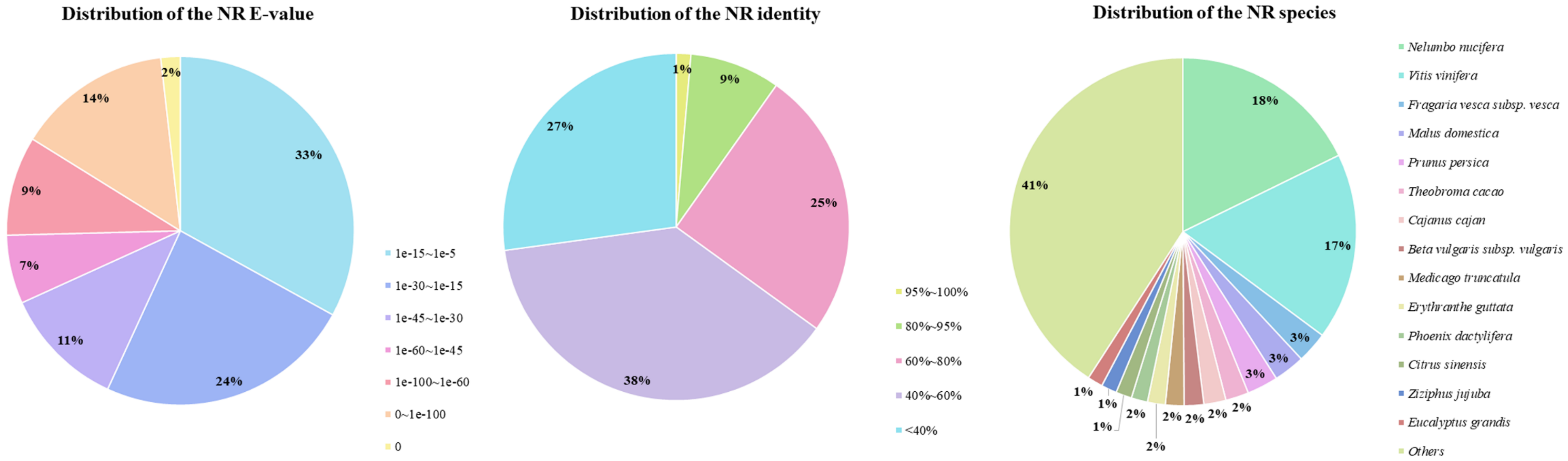

(C) Go classification of unigenes

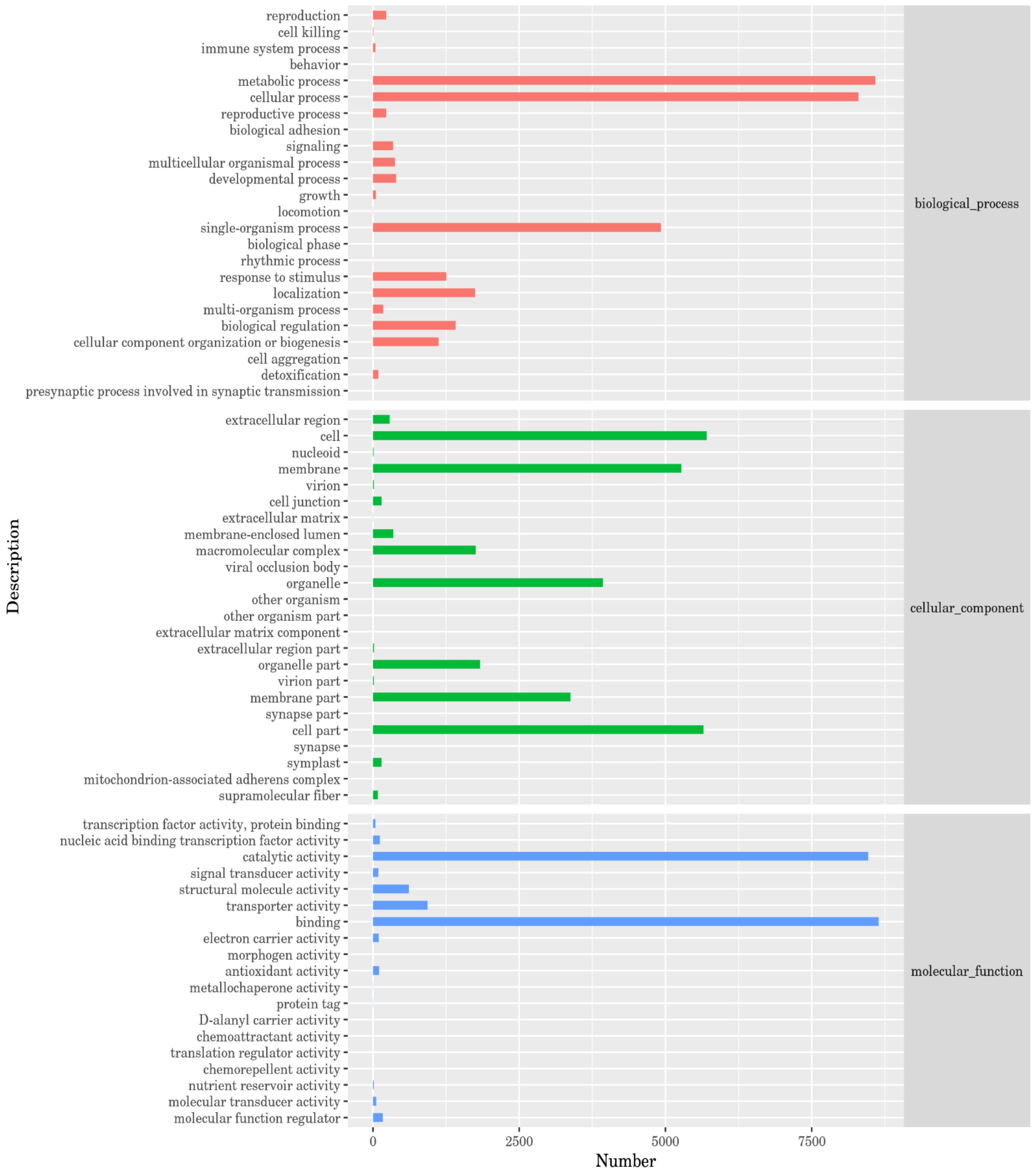

(D) KEGG classification of unigenes

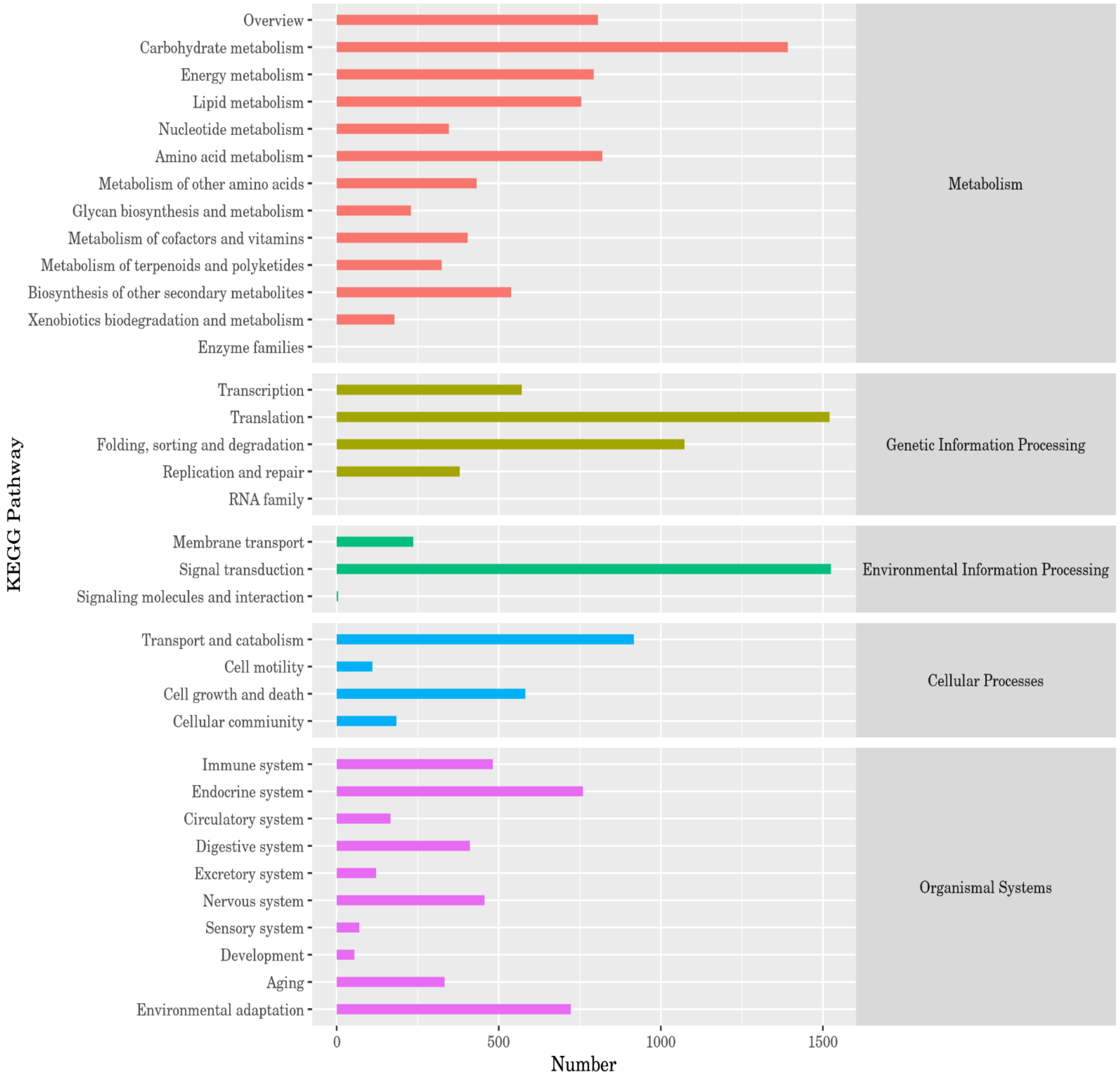

Supplement: Supplementary file 2 — Additional file 2. Supplementary Fig. S2. Functional annotation of unigenes in Clematis ‘Vyvyan Pennell’. [file 12870_2023_4696_MOESM2_ESM.pdf]
