# Supplementary material for: Seasonal variation of two floral patterns in Clematis ‘Vyvyan Pennell’ and its underlying mechanism
Source: BMC Plant Biol. 2024 Jan 2;24:22. doi: 10.1186/s12870-023-04696-9 (PMC10759560; doi:10.1186/s12870-023-04696-9)

Supplementary Fig.S3 GO and KEGG enrichment of the DEGs in compared group between stamens and petaloidy

(A) GO enrichment of 4 contrast groups

① Stage 5

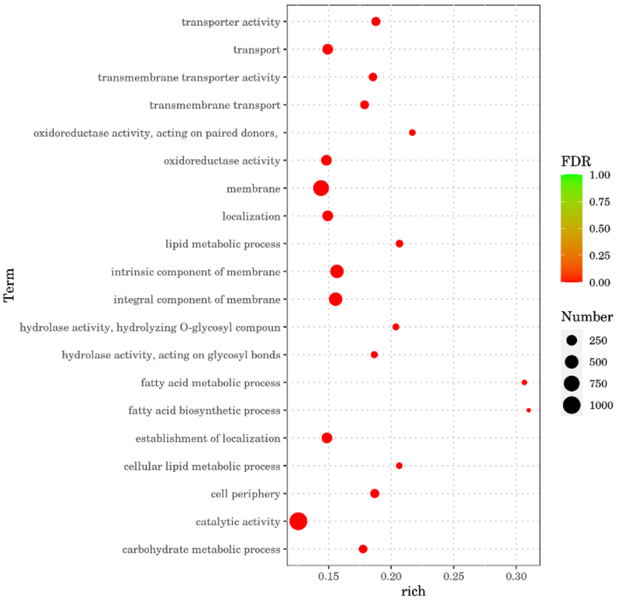

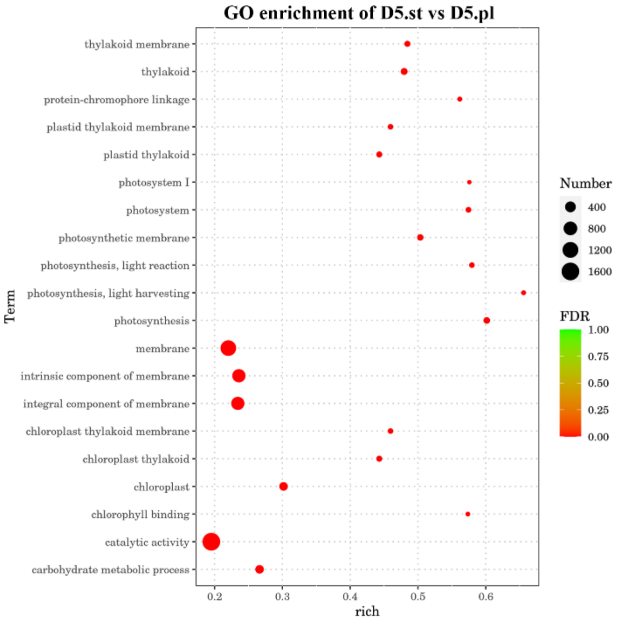

② Stage 7

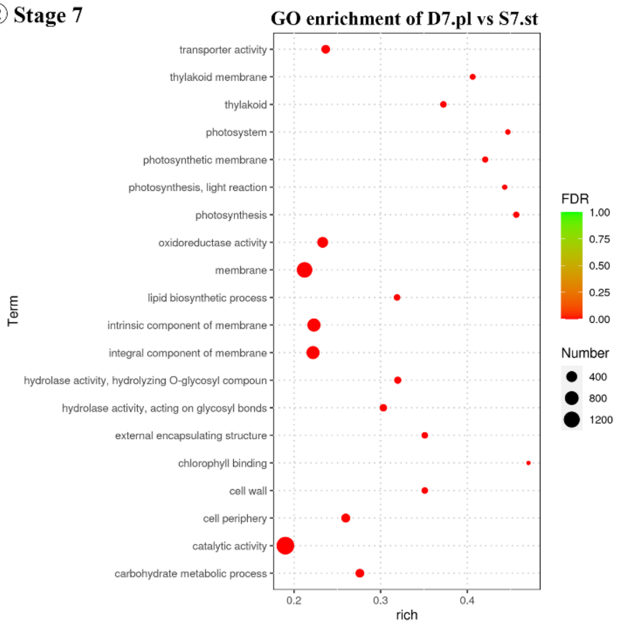

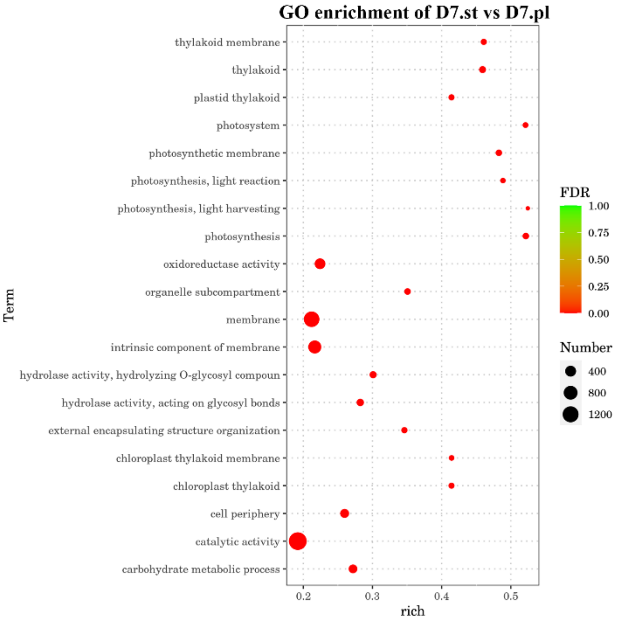

(B) KEGG enrichment of 4 contrast groups

① Stage 5

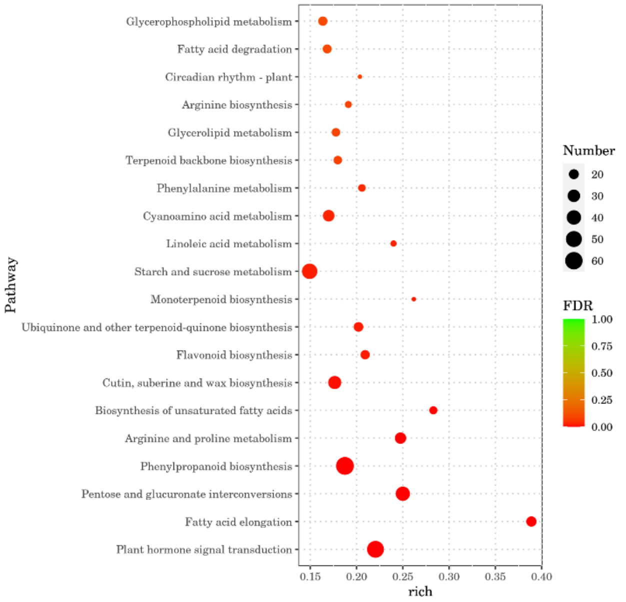

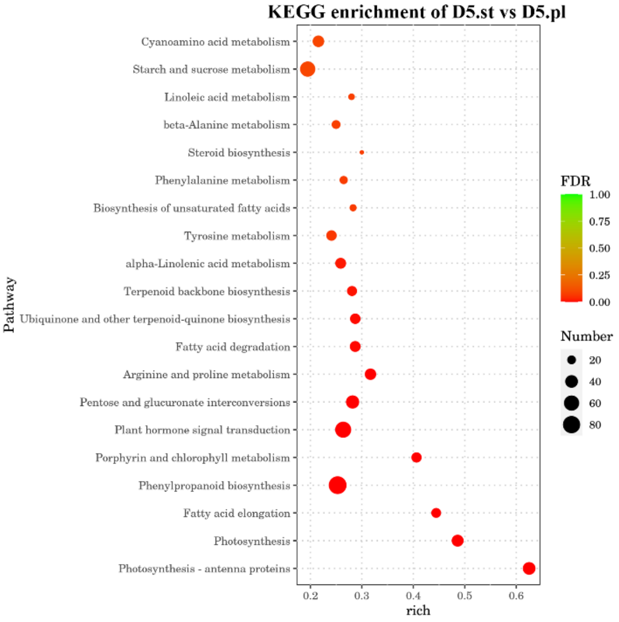

② Stage 7

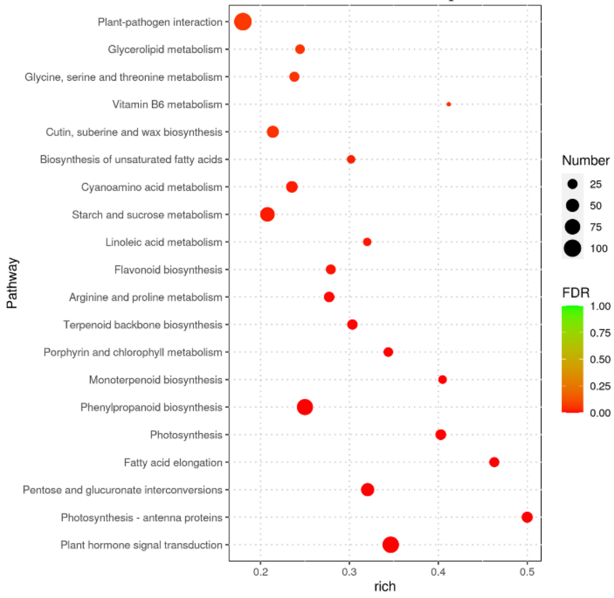

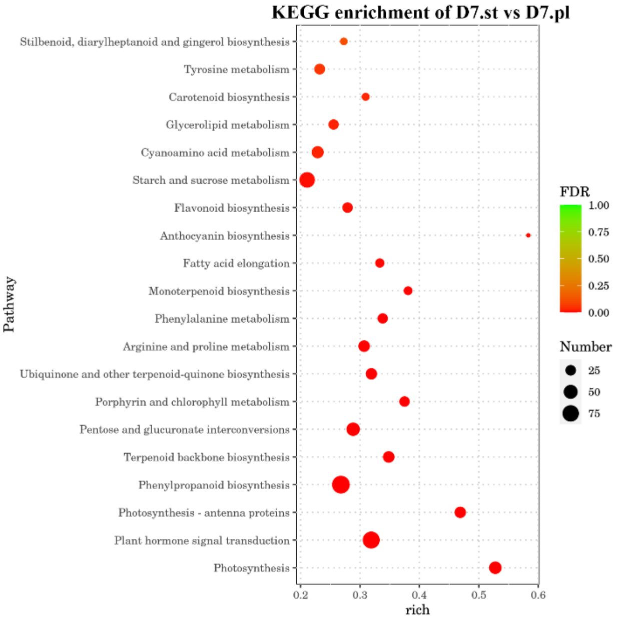

Supplement: Supplementary file 3 — Additional file 3: Supplementary Fig. S3. GO and KEGG enrichment of the DEGs in compared group between stamens and petaloidy. [file 12870_2023_4696_MOESM3_ESM.pdf]
