# Supplementary material for: Seasonal variation of two floral patterns in Clematis ‘Vyvyan Pennell’ and its underlying mechanism
Source: BMC Plant Biol. 2024 Jan 2;24:22. doi: 10.1186/s12870-023-04696-9 (PMC10759560; doi:10.1186/s12870-023-04696-9)

Supplementary Fig.S4 The content of plant hormone with no significant difference between seasonal organs in *Clematis* 'Vyvyan Pennell'

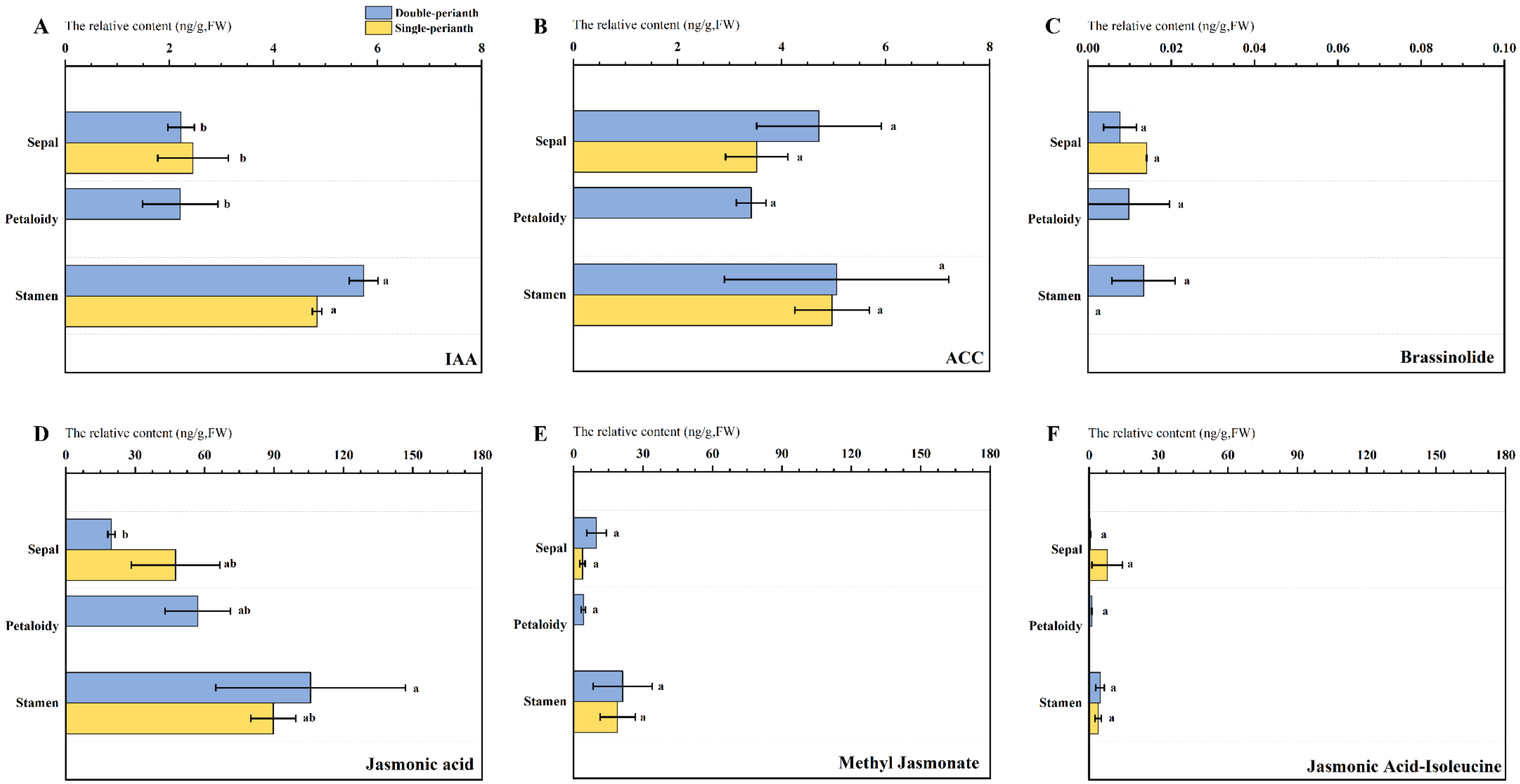

Supplement: Supplementary file 4 — Additional file 4: Supplementary Fig. S4. The content of plant hormone with no significant difference between seasonal organs in Clematis ‘Vyvyan Pennell’. [file 12870_2023_4696_MOESM4_ESM.pdf]
