# Supplementary material for: Seasonal variation of two floral patterns in Clematis ‘Vyvyan Pennell’ and its underlying mechanism
Source: BMC Plant Biol. 2024 Jan 2;24:22. doi: 10.1186/s12870-023-04696-9 (PMC10759560; doi:10.1186/s12870-023-04696-9)

Supplementary Fig.S5 Soft threshold value, modules, eigengenes expression pattern in yellow of WGCNA

(A) Soft threshold to conducted the scale-free network

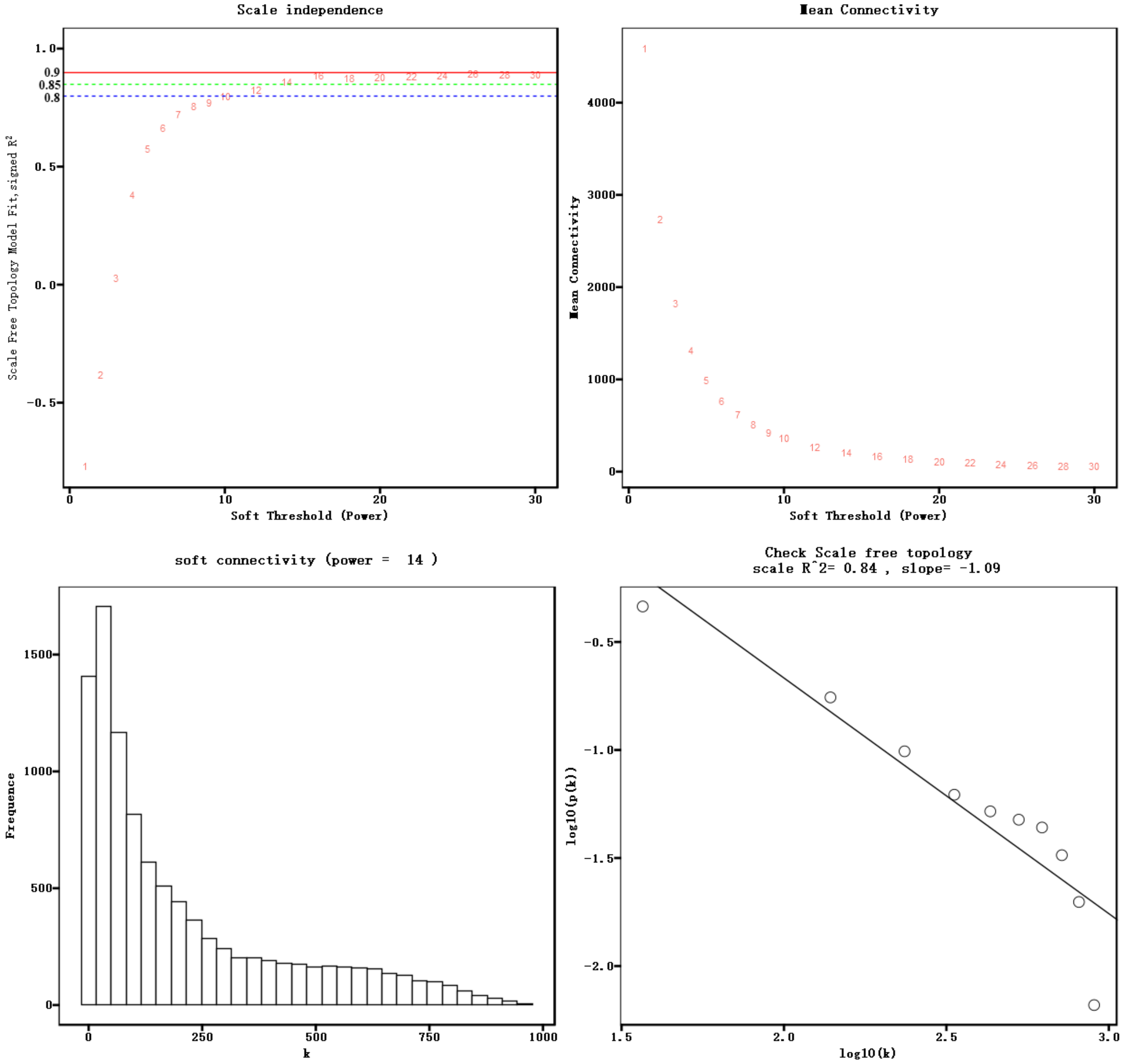

(B) The divided modules

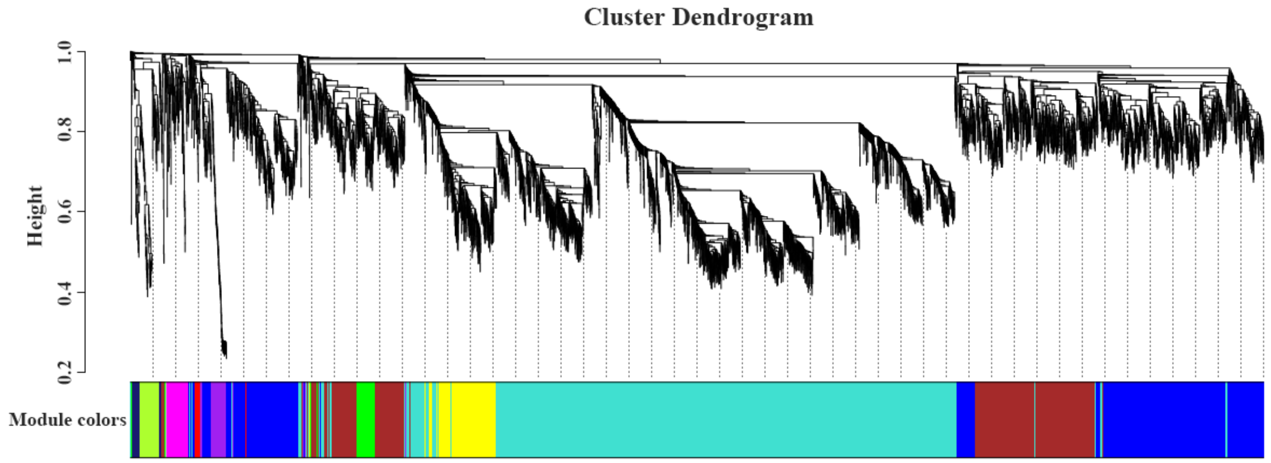

(C) The eigengenes expression pattern in yellow module

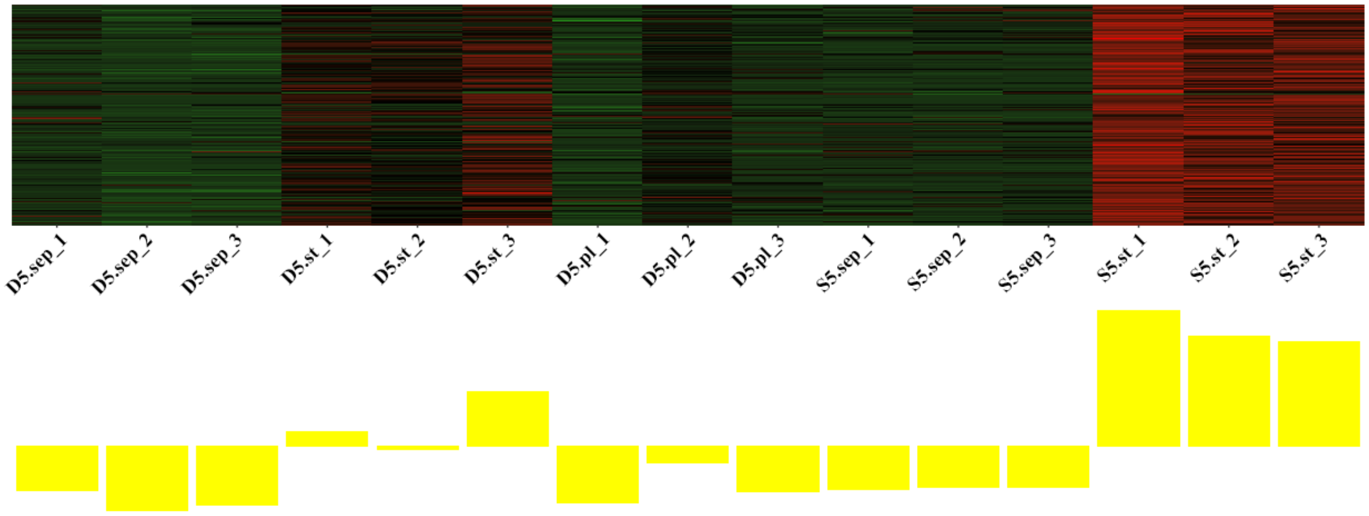

Supplement: Supplementary file 5 — Additional file 5: Supplementary Fig. S5. Soft threshold value, modules, eigengenes expression pattern in yellow of WGCNA. [file 12870_2023_4696_MOESM5_ESM.pdf]
