# Supplementary material for: Seasonal variation of two floral patterns in Clematis ‘Vyvyan Pennell’ and its underlying mechanism
Source: BMC Plant Biol. 2024 Jan 2;24:22. doi: 10.1186/s12870-023-04696-9 (PMC10759560; doi:10.1186/s12870-023-04696-9)
Supplement: Supplementary file 6 — Additional file 6: Supplementary Table. S2 Primers of floral-organ identity genes. [file 12870_2023_4696_MOESM6_ESM.pdf]

Supplementary Table.S2 Primers of floral-organ identity genes

(A) Primers for RT-qPCR

No.	genes ID	sequences for RT-qPCR	bp	products
1	qACTIN-F	ATATCCTCCGTTTGGACCTTG	21	101
	qACTIN-R	TTCCCGTTCTGCTGTCGTT	19	
2	qAP1/FUL-F	TCCTTCCTTCCAGTGCAGAC	20	115
	qAP1/FUL-R	TGTGGCGAAGCATCCAATGT	20	
3	qAP2-F	TGGGGGAGATCAGTTTGGTG	20	141
	qAP2-R	ACATGAACTGTTGAGGCTGGG	21	
4	qAP3-1-F	AACTGGCAACTGATGGTGCT	20	115
	qAP3-1-R	GCCAATCGGAGCTGATGGAA	20	
5	qAP3-2-F	GAGGGAGATACATCGTGCCAT	21	154
	qAP3-2-R	GGGTCAAAGCATAGGAGCCAT	21	
6	qPI-1-F	CAGGAGCAGCAAATGGAAATGA	22	107
	qPI-1-R	TGGATTGGCTGGACACGGAA	20	
7	qPI-2-F	GGACAGTGCACCAGCAAGAA	20	127
	qPI-2-R	GGTTTGGCTGGATGGACACA	20	
8	qAG-F	GCCTGATGCCTGGGAATGAA	20	132
	qAG-R	ACCCAAGTTGAAGCGTTGTTT	21	

(B)Primers for RT-PCR

No.	Genes ID	Sequences for RT-PCR	bp	products
1	actin-F	TTGCCATTCAGGCTGTTCTT	20	221
	actin-R	TTCCCGTTCTGCTGTCGTT	19	
2	AP1/FUL-F	ATGGGGAGAGGTAGGGTTCA	20	731
	AP1/FUL-R	GCTGAAAACTCTCACACCCTACT	23	
3	AP2-F	GTTCATCTCATCTCATGAAAAAGT	24	1517
	AP2-R	TCGCCTTCTCAAGTATGTGG	20	
4	AP3-1-F	GTCCATTTCCACTCAAGCCA	20	687
	AP3-1-R	ACAAAAATTAAGATGGGAAGAGGA	24	
5	AP3-2-F	TTCAAGCAAGGGTCAAAGCA	20	675
	AP3-2-R	AGAAGCATGGGTAGGGGGAA	20	
6	PI-1-F	GATTTCCCTATTTACTCTCCTGT	23	644
	PI-1-R	GAAAAGAGATCATGGGGAGAGG	22	
7	PI-2-F	AGATCATGGGGAGAGGAAAG	20	640
	PI-2-R	GTAGTTCTATTTGTTCTCCTGT	22	
8	AG-F	GGGGAGAGGCAAGATTGAGAT	21	818
	AG-R	CCCAAGTTGAAGGGTTGTTTGA	22	
