# Supplementary material for: Seasonal variation of two floral patterns in Clematis ‘Vyvyan Pennell’ and its underlying mechanism
Source: BMC Plant Biol. 2024 Jan 2;24:22. doi: 10.1186/s12870-023-04696-9 (PMC10759560; doi:10.1186/s12870-023-04696-9)
Supplement: Supplementary file 7 — Additional file 7: Supplementary Table.S3. Quality control and summary of the transcriptome sequence. [file 12870_2023_4696_MOESM7_ESM.pdf]

Supplementary Table.S3 Quality control and summary of the transcriptome sequence

(A) Sequencing data and quality control

Sample	Raw Reads	Bases (Gb)	Clean Reads	Clean Data (Gb)	Clean Reads (%)	N (%)	Q20 (%)	Q30 (%)
Ab_1	46593246	6.9889869	42080794	6.3121191	90.31	0.001393	96.55	91.28
Ab_2	44995750	6.7493625	40774578	6.1161867	90.61	0.001421	96.6	91.36
Ab_3	42430870	6.3646305	38537232	5.7805848	90.82	0.001394	96.54	91.28
Asep_1	40634560	6.095184	36748658	5.5122987	90.43	0.001399	96.39	91.11
Asep_2	46168412	6.9252618	42061638	6.3092457	91.1	0.001078	96.36	91.07
Asep_3	45822656	6.8733984	41694866	6.2542299	90.99	0.001412	96.42	91.21
Ast_1	45908184	6.8862276	42015682	6.3023523	91.52	0.001419	96.54	91.29
Ast_2	40757082	6.1135623	37428464	5.6142696	91.83	0.001386	96.49	91.16
Ast_3	40511290	6.0766935	37358716	5.6038074	92.21	0.001407	96.32	90.88
Apl_1	46043832	6.9065748	41393414	6.2090121	90.00	0.001408	96.56	91.37
Apl_2	44297230	6.6445845	40477054	6.0715581	91.37	0.001406	96.47	91.25
Apl_3	50400638	7.5600957	45804922	6.8707383	90.88	0.001391	96.29	90.82
Bb_1	43286932	6.4930398	39091754	5.8637631	90.3	0.001547	96.22	90.74
Bb_2	39910576	5.9865864	36338602	5.4507903	91.05	0.001384	96.36	91.1
Bb_3	43146222	6.4719333	39502960	5.925444	91.55	0.001547	96.42	91.21
Bsep_1	44106692	6.6160038	40401982	6.0602973	91.6	0.001417	96.39	91.2
Bsep_2	44538460	6.680769	40514388	6.0771582	90.96	0.001376	96.23	90.93
Bsep_3	50673364	7.6010046	46535220	6.980283	91.83	0.001379	96.4	91.11
Bst_1	40762672	6.1144008	37324164	5.5986246	91.56	0.001387	96.24	90.89
Bst_2	44174480	6.626172	40472998	6.0709497	91.62	0.001407	96.02	90.41
Bst_3	42327574	6.3491361	38875718	5.8313577	91.84	0.001389	96.26	90.78
Bpl_1	44437534	6.6656301	40946196	6.1419294	92.14	0.001374	96.17	90.7
Bpl_2	40762982	6.1144473	37072244	5.5608366	90.94	0.001403	95.83	90.21
Bpl_3	46266022	6.9399033	42710150	6.4065225	92.31	0.001379	96.37	91.13
Cb_1	43432732	6.5149098	40000402	6.0000603	92.09	0.001407	96.08	90.59
Cb_2	43121708	6.4682562	39192676	5.8789014	90.88	0.001267	96.72	91.76
Cb_3	41609908	6.2414862	38224312	5.7336468	91.86	0.001421	96.4	91.12
Csep_1	40899896	6.1349844	37248632	5.5872948	91.07	0.001408	96.33	91.05
Csep_2	42850522	6.4275783	39190760	5.878614	91.45	0.001402	96.45	91.26
Csep_3	45612336	6.8418504	41906526	6.2859789	91.87	0.00141	96.14	90.68
Cst_1	45144062	6.7716093	40992164	6.1488246	90.8	0.001089	96.51	91.2
Cst_2	52279730	7.8419595	47950926	7.1926389	91.71	0.001412	96.62	91.37
Cst_3	48921446	7.3382169	43944292	6.5916438	90.00	0.00139	95.36	89.01
Db_1	42324018	6.3486027	38432098	5.7648147	90.8	0.001396	96.42	91.18
Db_2	43858600	6.57879	39883224	5.9824836	90.93	0.001401	96.43	91.21
Db_3	49365632	7.4048448	44632704	6.6949056	90.41	0.001424	96.45	91.16
Dsep_1	45795656	6.8693484	41750822	6.2626233	91.16	0.00138	96.34	90.97
Dsep_2	42903200	6.43548	38814082	5.8221123	90.46	0.00139	96.5	91.29
Dsep_3	42951208	6.4426812	38543840	5.781576	89.73	0.001393	96.43	91.19
Dst_1	45297904	6.7946856	41436052	6.2154078	91.47	0.001402	96.45	91.09
Dst_2	48527874	7.2791811	43697994	6.5546991	90.04	0.001395	96.57	91.36
Dst_3	47739772	7.1609658	43367832	6.5051748	90.84	0.00141	96.5	91.23

Clean Reads %: the percentage of high-quality reads in sequenced reads.

N (%): the percentage of fuzzy bases.

(B) Summary of transcripts and unigenes

	Transcript	Unigene
Total Length (bp)	515541864	178829438
Sequence Number	464907	209056
Max. Length (bp)	28769	28769
Mean Length (bp)	1108.91	855.41
N50 (bp)	1712	1158
N50 Sequence No.	89762	39604
N90 (bp)	455	380
N90 Sequence No.	324864	155989
GC%	40.72	40.36
