# Supplementary material for: Seasonal variation of two floral patterns in Clematis ‘Vyvyan Pennell’ and its underlying mechanism
Source: BMC Plant Biol. 2024 Jan 2;24:22. doi: 10.1186/s12870-023-04696-9 (PMC10759560; doi:10.1186/s12870-023-04696-9)
Supplement: Supplementary file 8 — Additional file 8: Supplementary Table.S4. The Log2(Foldchange) of 43 DEGs in MADS family. [file 12870_2023_4696_MOESM8_ESM.pdf]

Supplementary Table.S4 The Log_2(Foldchange) of 43 DEGs in MADS family

		Log2(Foldchange)											
**ID**	**Annotation**	**Ab VS Cb**	**Bb VS Dd**	**Asep VS Csep**	**Bsep VS Dsep**	**Ast VS Cst**	**Bst VS Dst**	**Apl VS Cst**	**Bpl VS Dst**	**Asep VS Apl**	**Bsep VS Bpl**	**Ast VS Apl**	**Bst VS Bpl**
TRINITY_DN3196_c0_g1	MADS-box protein SOC1	0.29	-0.76	-1.21**	-0.88	-0.10	-0.13	1.44**	1.17**	-0.30	0.05	-1.56**	-1.33**
TRINITY_DN27432_c0_g1	MADS-box protein SOC1	0.48	-0.47	-0.56	0.34	-0.32	0.99	2.22*	3.24**	-1.15	0.31	-2.50*	-2.26
TRINITY_DN44234_c0_g1	MADS-box protein SOC1	-0.27	-2.13	1.03	0.37	-0.24	-0.73	0.26	3.35**	2.38	0.00	-0.50	-4.05**
TRINITY_DN23187_c0_g1	MADS-box protein SVP	1.67*	-0.64	-3.40**	-3.18**	1.19	0.17	1.01	0.00	-2.09**	-1.44**	0.17	0.14
TRINITY_DN10698_c0_g1	Agamous-like MADS-box protein AGL19	-0.25	0.29	0.95	1.18**	0.40	-0.34	0.24	0.37	0.07	0.18	0.14	-0.74
TRINITY_DN26638_c0_g1	Floral homeotic protein APETALA 1(AP1)	-0.47	-0.06	1.28	0.08	-1.46*	-0.78	-0.09	0.65	0.45	-0.03	-1.38	-1.46*
TRINITY_DN26638_c1_g1	Floral homeotic protein APETALA 1(AP1)	-0.96	-1.83*	-1.12	-1.73	0.00	-0.44	-1.52	-1.22	-0.85	-0.78	1.45	0.79
TRINITY_DN29580_c0_g1	Floral homeotic protein APETALA 1(AP1)	-1.20	0.32	0.20	0.84	0.65	0.68	-4.12**	-2.39**	0.22	0.30	4.75**	3.04**
TRINITY_DN19683_c0_g2	Agamous-like MADS-box protein AGL8	-0.45	-0.01	0.84	0.00	-0.38	0.38	1.53	2.28*	0.32	0.00	-1.88	-1.89
TRINITY_DN19683_c0_g1	Agamous-like MADS-box protein AGL8	-1.33	-0.39	-0.95	-0.92	-2.02*	-2.60*	0.19	-0.74	-0.82	-0.38	-2.20	-1.76
TRINITY_DN4547_c0_g1	Agamous-like MADS-box protein AGL8	-1.35*	-0.93	-2.28**	-2.00**	-1.58**	-1.63**	-0.70	-1.31**	0.00	-0.04	-0.91	-0.36
TRINITY_DN6305_c0_g1	Floral homeotic protein MADS 1(MADS1)	0.30	0.05	0.05	-1.30	1.12**	0.67	3.19**	3.47**	6.24**	5.32**	-2.09**	-2.84**
TRINITY_DN10077_c0_g1	MADS-box transcription factor 4(MADS4)	0.86	0.49	0.48	2.31**	1.44**	0.95	2.73**	2.57**	-0.21	1.53**	-1.31*	-1.65**
TRINITY_DN7195_c0_g1	MADS-box transcription factor 16(MADS16)	1.31*	0.42	-1.05	0.25	1.19**	0.83	2.81**	2.57**	-0.75	-0.11	-1.65*	-1.78**
TRINITY_DN194671_c0_g1	MADS-box transcription factor 2(MADS2)	2.13*	0.23	0.52	1.45*	1.08	2.10**	1.07	1.75**	-0.32	-0.13	0.00	0.31

TRINITY_DN6189_c0_g1	MADS-box transcription factor 2(MADS2)	1.42*	1.14**	-0.01	0.78	1.06**	0.98	0.92	0.74	-0.85	-0.10	0.12	0.21
TRINITY_DN32932_c0_g1	MADS-box transcription factor 2(MADS2)	1.35*	1.35**	-0.44	0.80	1.40**	1.60**	0.73	0.74	-0.65	-0.24	0.65	0.81
TRINITY_DN3972_c2_g1	Floral homeotic protein AGAMOUS AG	0.68	-0.38	-0.32	0.87	1.06**	0.96	3.85**	4.67**	6.12**	7.04**	-2.81**	-3.74**
TRINITY_DN19875_c0_g1	Agamous-like MADS-box protein AGL6	0.58	1.38**	-0.87	-0.27	1.35**	0.30	-1.65**	-2.59**	-1.03*	-0.94	2.97**	2.86**
TRINITY_DN33356_c0_g1	MADS-box transcription factor 6(MADS6)	1.35	0.82	-0.22	-0.03	1.62*	0.13	-1.16	-2.65**	-0.76	-0.23	2.74	2.74**
TRINITY_DN60875_c0_g1	MADS-box transcription factor 6(MADS6)	-0.65	1.12	0.10	-0.25	0.78	0.77	-1.62	-2.22*	-0.12	-0.40	2.36**	2.93**
TRINITY_DN774_c0_g3	MADS-box transcription factor 6(MADS6)	1.12	1.40**	-0.64	-0.14	1.61**	0.62	-1.03	-2.41**	-1.21*	-0.59	2.61**	3.00**
TRINITY_DN216754_c0_g1	Agamous-like MADS-box protein AGL80	0.86	-0.61	0.00	0.34	-1.94	-1.76	0.00	1.18	0.00	0.00	-1.91	-2.89*
TRINITY_DN11025_c0_g1	Agamous-like MADS-box protein AGL80	1.01	-1.78**	0.35	-0.10	-2.26**	-2.32	3.13**	2.49**	0.27	0.35	-5.35**	-4.83*
TRINITY_DN2699_c0_g1	Agamous-like MADS-box protein AGL80	1.11	-3.70**	1.78	-0.31	-1.84*	-3.84**	1.95	3.96**	3.92	-0.32	-3.80**	-7.78**
TRINITY_DN7766_c0_g1	Agamous-like MADS-box protein AGL80	5.15*	-3.70**	-0.32	-0.50	-0.72	-0.58	7.30**	5.77**	-1.49	0.39	-8.00**	-6.39
TRINITY_DN14411_c0_g1	Agamous-like MADS-box protein AGL61	-0.21	1.09	1.17	2.66*	-0.30	-1.47	0.98	0.12	-0.11	1.10	-1.27	-1.59
TRINITY_DN11675_c0_g1	Agamous-like MADS-box protein AGL61	3.74**	0.83	-1.00	0.69	-0.26	0.29	2.28**	1.09**	-1.65	0.46	-2.55**	-0.83
TRINITY_DN8024_c0_g2	Agamous-like MADS-box protein AGL61	3.80**	0.20	-1.76	-0.62	-0.77	-0.48	1.99*	2.13**	-0.72	-1.36	-2.77**	-2.64*
TRINITY_DN162337_c0_g1	Agamous-like MADS-box protein AGL62	-0.23	-1.53	0.61	0.51	-0.70	-3.15**	0.85	-0.01	0.53	-0.48	-1.55	-3.07**
TRINITY_DN57701_c0_g1	Agamous-like MADS-box protein AGL62	-1.27	-0.59	0.36	0.00	-0.71	-2.83**	0.85	0.35	0.57	0.00	-1.53	-3.09**

TRINITY_DN39436_c0_g1	Agamous-like MADS-box protein AGL62	2.18	-1.00	0.00	0.00	-2.01**	-1.46	0.80	2.06	0.97	0.00	-2.79**	-3.48**
TRINITY_DN4517_c0_g1	Agamous-like MADS-box protein AGL62	1.45	-2.28	1.10	-0.31	-0.82	-3.14**	3.48**	1.85	1.29	-0.32	-4.27**	-4.94**
TRINITY_DN9579_c0_g1	Agamous-like MADS-box protein AGL62	3.31**	-0.69	0.84	0.00	-0.32	0.31	2.28**	5.09**	2.22	0.31	-2.60**	-4.77**
TRINITY_DN14411_c0_g2	Agamous-like MADS-box protein AGL23	0.16	-2.76**	1.16	-1.28	-1.40**	-5.46**	1.61	1.11	3.88	-1.30	-3.01**	-6.51**
TRINITY_DN56919_c0_g1	Agamous-like MADS-box protein AGL82	-0.23	-0.02	0.00	0.00	-1.27	-0.36	1.86	2.36	0.31	0.00	-3.09**	-2.69*
TRINITY_DN611_c1_g1	Agamous-like MADS-box protein AGL92	0.71	-1.15	0.92	-0.11	-1.10	-2.28*	1.89	2.92**	3.52	0.34	-2.99**	-5.22**
TRINITY_DN20041_c0_g1	Agamous-like MADS-box protein AGL103	0.57	-1.23	0.65	0.34	-1.77**	-2.66**	0.69	1.77	2.95	0.31	-2.45**	-4.40**
TRINITY_DN81428_c0_g1	Agamous-like MADS-box protein AGL104	5.46**	0.43	0.00	-0.92	-1.58	1.58	0.41	1.79	0.00	3.49**	-1.94	-0.24
TRINITY_DN30709_c1_g1	Agamous-like MADS-box protein AGL15	1.52	0.51	-0.92	-0.02	1.93**	1.61**	3.79**	4.54**	2.77	2.51*	-1.88*	-2.96**
TRINITY_DN75841_c0_g1	MADS-box transcription factor 57	-0.43	0.72	-1.09	-0.47	-0.92	0.83	0.60	-0.18	0.12	0.01	-1.53*	0.96
TRINITY_DN2562_c0_g1	Agamous-like MADS-box protein AGL21	-0.47	-0.29	0.67	1.25**	0.15	-0.25	-0.22	0.77	0.62	0.12	0.35	-1.06*
TRINITY_DN23006_c0_g1	MADS-box protein GGM13	1.48	-2.55**	0.00	0.00	-0.15	0.60	1.86	3.65**	0.32	0.00	-1.97	-3.03**

Note: $|log2FC| > 1$;* represents significant difference, $p < 0.05$; ** represents extremely significant difference, $p < 0.01$
